# Effect of PTPN22, FAS/FASL, IL2RA and CTLA4 genetic polymorphisms on the risk of developing alopecia areata: A systematic review of the literature and meta-analysis

**DOI:** 10.1371/journal.pone.0258499

**Published:** 2021-11-04

**Authors:** S. R. Gil-Quiñones, I. T. Sepúlveda-Pachón, G. Sánchez Vanegas, L. D. Gutierrez-Castañeda

**Affiliations:** 1 Clinical Epidemiology Program, Fundación Universitaria de Ciencias de la Salud (FUCS), Bogotá, Colombia; 2 Clinical Epidemiology Program, Research Institute, Fundación Universitaria de Ciencias de la Salud (FUCS), Bogotá, Colombia; 3 Research Institute, Group of Basic Sciences in Health (CBS)-FUCS, Fundación Universitaria de Ciencias de la Salud (FUCS), Bogotá, Colombia; Unicamillus, Saint Camillus International University of Health Sciences, ITALY

## Abstract

**Objectives:**

Genetic association studies on alopecia areata (AA) performed in various populations have shown heterogeneous results. The aim of the current review was to synthesize the results of said studies to estimate the impact of *FAS*, *FASL*, *PTPN22*, *CTLA4* and *IL2RA* gene polymorphisms on AA susceptibility.

**Design:**

A systematic literature search was conducted in the Medline, Web of Science, Scopus, EMBASE and LILACS databases. Studies published up to June 2020 were included. The results available in the grey literature including the Open Grey and Google Scholar databases were also used. The texts of potentially related studies were screened by individual reviewers. Evidence of publication bias was assessed using the Newcastle-Ottawa scale and the quality of evidence was assessed using the GRADE system. The quantitative synthesis was performed using the fixed effect model.

**Results:**

Out of 1784 articles, we identified 18 relevant articles for the qualitative synthesis and 16 for the quantitative synthesis. In a study of rs2476601 polymorphism of *PTPN22* gene, including 1292 cases and 1832 controls, a correlation was found with the risk of developing AA in the allelic model (OR1.49 [95% C:1.13–1.95]), the heterozygous codominant (OR1.44 [95% CI:1:19–1.76]) and dominant model (OR1.43 [95% CI:1.18–1.73]). No association was found between the presence of *FASL*, *PTPN22*, *CTLA* and *IL2RA* gene polymorphisms with AA susceptibility.

**Conclusions:**

The results suggest that the *T* allele of the single nucleoid polymorphism (SNP) rs2476601 in *PTPN22* gene is a risk factor for developing alopecia areata. However, more robust studies defining the ethnic background of the population of origin are required, so that the risk identified in the present study can be validated. Additionally, a greater number of studies is necessary to evaluate the role of the *FAS*, *FASL*, *PTPN22*, *CTLA4* and *IL2RA* genetic variants, given the heterogenous results found in the literature.

## Introduction

Alopecia areata (AA) is a multifactorial disease in which environmental, neuro-endocrinological, immunological and genetic factors are involved [1]. AA affecting the hair follicle is the most common form, through a breakdown in immune privilege of the hair follicle in the anagen (hair growth) phase by CD4^+^ and CD8^+^ T lymphocytes, resulting in non-scarring hair loss [2]. AA encompasses a spectrum of disease patterns including patchy alopecia (diffuse loss) alopecia totalis (total hair loss on the scalp) and alopecia universalis (loss of hair on the entire body) [3].

Given the broad genetic component of AA, the effect of several immune response modulator genes has been discussed [4]. Genome-wide association studies (GWAS) have revealed the involvement of genes related to innate and adaptive immunity. These population studies have proposed that the *PTPN22*, *FAS*, *FASL*, *IL2RA* and *CTLA4* genes are related to the risk of developing AA [5].

The *FAS* gene encodes tumor necrosis factor receptor superfamily member 6 and *FASL* gene encodes tumor necrosis factor ligand superfamily member 6, located on chromosomes 10 and 1 respectively. Their function is critical in immunological homeostasis given their ability to induce cell death and proliferation or differentiation of T lymphocytes [6]. The *PTPN22* gene is located on chromosome 1 and encodes a protein involved in T lymphocyte signaling, downregulating the T cell receptor and the production of type 1 interferon [7]. *CTLA4* gene encodes a lymphocyte receptor that promotes T-cell anergy, preventing autoimmune reactions [8]. Finally, *IL2RA* is located on chromosome 10 and encodes one of the subsets of the IL-2 receptor, which is involved in the regulation of immunological tolerance and the control of regulatory T cells [9].

Several studies have reported an association between the aforementioned genes and the risk of developing AA. A case-control study carried out by Kalkan et al., which investigated *FAS-*670*A/G* (rs1800682) and *FASL*-124*A/G* (rs5030772) polymorphisms in the Turkish population, found that the GG genotype of rs1800682 polymorphism was a protective factor against AA with a reduced risk of AA compared with the *AA* and *AG* genotypes (OR 0.07 [95% CI: 0.00–0.41]) [10]. The *PTPN22* gene and the *CT* genotype of rs2476601 polymorphism, were associated with AA susceptibility (OR 3.31 [95% CI: 1.008–10.892]) [11]. A haplotype analysis of *CTLA-4* determined that the presence of alleles A (rs231775) and G (*CT*60: rs3087243) is associated with a lower risk of the disease (OR 0.28 [95% CI: 0.09–0.82]) [12]. An association analysis of the *IL2RA* gene in the Chinese population, determined that the prevalence of rs3118470 polymorphism genotype in the AA group was 48.2% for *T/C*, 35.6% for *T/T*, and 16.2% for *C/C*. No Odds Ratio risk estimator was calculated [13].

The results of primary studies of genetic association between polymorphisms in said genes with AA susceptibility in different populations, have yielded heterogeneous and sometimes contradictory results. For these reasons, the present study synthesizes the available evidence regarding *PTPN22*, *FAS*, *FASL*, *IL2RA* and *CTLA4* gene polymorphisms and their correlation with AA susceptibility.

## Methods

### Inclusion and exclusion criteria

This review was based on the PRISMA guidelines (S1 File). Studies should satisfy the following criteria: a) Human population studies; b) Genetic association studies on AA risk and *FAS/FASL*, *PTPN22*, *CTLA4* and *IL2RA* gene polymorphisms; c) Studies in patients diagnosed with any type of AA (patches/totalis/universalis). The studies should be genome-wide associated studies (case-control/GWAS case-control studies). The search was conducted without language or year of publication restrictions.

The exclusion criteria were: control patients presenting with an autoimmune diseases (since their presence may alter the interpretation of the effect of polymorphism on AA susceptibility) and studies excluded for ethical concerns or distortion of scientific results.

### Search strategy

An exhaustive search conducted in the Medline, Web of Science, Scopus, EMBASE and LILACS databases by combining search strategies using the PICO elements of the research question (for observational studies): “Gene (*FAS-FASL/PTPN22/CTLA4/IL2RA*)”, “Polymorphism / Genetic Variant” and “Alopecia areata”. Studies published up to June 2020 were included. A literature search was conducted for each gene and its homonyms. Other potentially useful resources were identified in Open Grey and Google Scholar. The complete search strategy is fully stated in S2 File. The records were downloaded to a reference software and duplicates were eliminated. Two authors independently selected eligible studies (SG/IS) by reading the title and abstract. Subsequently, the full text of potentially relevant articles was reviewed and meticulously examined for compliance with the inclusion and exclusion criteria using a pre-established form. Discrepancies found in these phases of the process, were resolved by discussion with consensus and checked by a third author (GS). The reasons for exclusion of studies were documented.

### Data extraction

Data was extracted by two authors (SG/IS) using a preestablished form, which included highly relevant information to develop the synthesis, such as: article reference, year of publication, number of cases and controls, Hardy-Weinberg equilibrium, clinical diagnosis, sequencing method, gene analyzed, allelic and genotypic frequency, clinical significance of the variant analyzed, source of funding and conflicts of interest. Authors were contacted via email to request missing information. Discrepancies in extracted data were resolved by a third author (LG).

### Assessment of risk of bias and quality of evidence for individual studies

The Newcasttle-Ottawa scale (NOS) scoring system (0–9) was used to determine the risk of bias for each individual study. Domains inherent to case-control studies, such as case/control selection, comparability, and exposure measurement were evaluated. In accordance with the trend in the scientific community for this type of study, a cut-off ≥7 was established to consider low risk of bias. The methodological quality of the primary studies was assessed using the NOS and the quality of the evidence found was established using the GRADE tool (). This evaluation was carried out by 2 authors (SG/IS) and discrepancies were resolved by a third author (GS).

### Statistical analysis

All statistical analyses were performed using Stata16^®^ and Revman V5.2 software. Testing for Hardy-Weinberg equilibrium was verified in the control group of each study, if not detected, this calculation was made using a chi-squared test (X^2^). Odds Ratio (OR) was calculated for dichotomous data using the 2x2 table. The summary measure of the effect was calculated as an Odds Ratio with its respective 95% confidence interval and p-value (a p value <0.05 is considered statistically significant).

The heterogeneity across the included studies was visually assessed based on the results of the forest plots, the chi-squared test for heterogeneity (X^2^) with a statistical significance value of 10%, the H^2^ test and the I-squared (I^2^) statistic. A fixed effect model assuming an I^2^ statistic of less than 50% was used for the quantitative synthesis. The random effect model was calculated, and subgroup analyses were performed when the I^2^ score was above 50%, considered to indicate substantial clinical heterogeneity. Heterogeneity was addressed, by performing subgroup analyses across studies according to the NOS score and control group selection (hospital or community-based controls). Meta-regression was applied to determine possible heterogeneity derived from the number of subjects included in the studies. When the heterogeneity value was greater than 75%, it was decided to exclude these studies from the meta-analysis.

## Results

### Results of search

**Fig 1:**
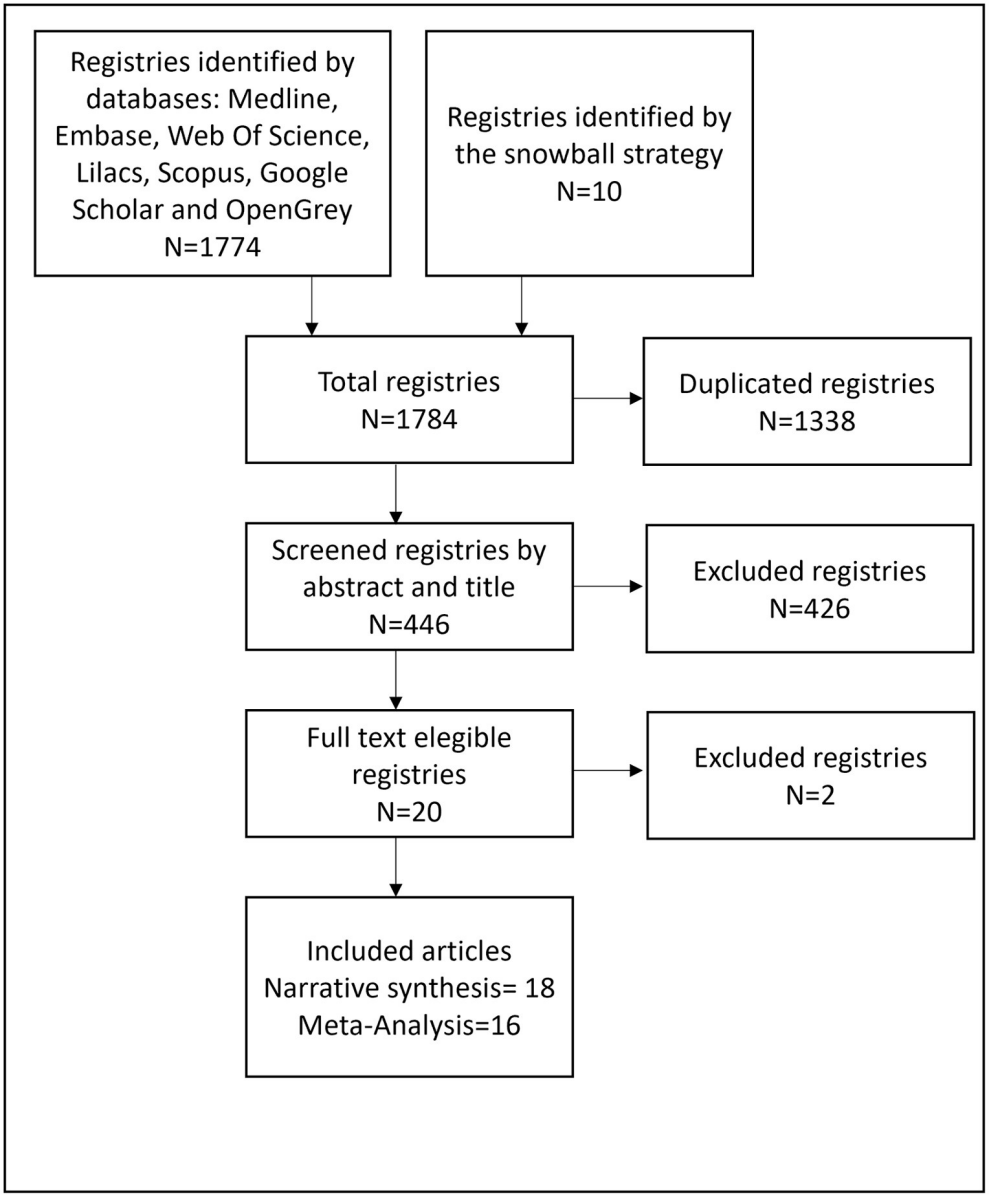
Study flow diagram.

The flow chart of literature search and screening is shown in Fig 1. By means of the combination of terms listed in the search strategy, carried out in the different databases, a total of 1774 articles were obtained, to which 10 articles regarding the application of the snowball strategy to relevant articles, were added. We identified and removed 1338 duplicate records from the 1784 records, obtaining 446 eligible articles for screening by title and abstract. According to the application of the selection criteria 426 irrelevant records were removed. Finally, 20 articles were included for assessment of their full text. Eighteen articles were included in the qualitative synthesis and 16 in the meta-analysis.

### Study characteristics

A total of 17 case-control studies and one case-control GWAS were included. Table 1 contains the characteristics of the included studies. The distribution of the studies according to the gene and genetic variants was: 7/18 for the PTPN22 gene (rs2476601), 4/18 for the *FAS* gene (rs1800682), 3/18 for the *FASL* gene (rs5030772), 3/18 for the *IL2RA* gene (rs3118470) and 4/18 for the *CTLA4* gene (rs231775). These studies were published between 2008 and 2020 and were conducted mainly in geographic regions of Europe and Asia, followed by North America and Mexico to a lesser extent. Control groups composition was community or control-based. The main genotyping method was PCR-RFLP (Polymerase chain reaction—Restriction fragment length polymorphisms). The NOS score ranged between 8 and 9. The specific scores for each domain of bias are listed in Table 2. The studies included the 3 types of alopecia: patchy, totalis and universalis. Allelic and genotypic frequencies are shown in Table 3.

**Table 1:** Characteristics of the studies included in the systematic review and meta-analysis.

First Author	Year	Gene	Variant	Geographic Region	Study design	Control origin	NOS*	#Cases	#Controls
Betz RC [14]	2007	*PTPN22*	rs2476601	Germany/Belgium	Case-control	Community	8	435	628
Bhanusali D [15]	2014	*PTPN22*	rs2476601	United States	Case-control	Community	8	365	273
El-Zawahry B [16]	2013	*PTPN22*	rs2476601	Egypt	Case-control	Community	9	103	100
Kemp E [17]	2006	*PTPN22*	rs2476601	England	Case-control	Unclear	8	196	507
Moravvej H [4]	2018	*PTPN22*	rs2476601	Iran	Case-control	Community	9	69	69
Salinas-Santander M [11]	2015	*PTPN22*	rs2476601	Mexico	Case-control	Community	9	64	225
Shehata W [18]	2020	*PTPN22*	rs2476601	Egypt	Case-control	Community	9	60	30
Fan X [19]	2010	*FAS*	rs1800682	China	Case-control	Hospital	8	84	84
Kalkan G [10]	2013	*FAS*	rs1800682	Turkey	Case-control	Hospital	8	118	118
Seleit I [20]	2018	*FAS*	rs1800682	Egypt	Case-control	Unclear	8	60	40
Tabatabaei-Panah P [21]	2020	*FAS*	rs1800682	Iran	Case-control	Hospital	8	60	60
Kalkan G [10]	2013	*FASL*	rs5030772	Turkey	Case-control	Hospital	8	118	118
Seleit I [20]	2018	*FASL*	rs5030772	Egypt	Case-control	Unclear	8	60	40
Tabatabaei-Panah P [21]	2020	*FASL*	rs5030772	Iran	Case-control	Hospital	8	60	60
Miao Y [13]	2013	*IL2RA*	rs3118470	China	Case-control	Hospital	8	427	430
Moravvej H [4]	2018	*IL2RA*	rs3118470	Irán	Case-control	Community	9	69	69
Redler S [22]	2012	*IL2RA*	rs3118470	Germany/Belgium	Case-control	Community	9	768	658
Ismail N [23]	2020	*CTLA4*	rs231775	Egypt	Case-control	Hospital	8	93	93
John K [24]	2011	*CTLA4*	rs231775	Central Europe	GWAS Case-control	Unclear	8	1196	1280
Megiorni F [12]	2013	*CTLA4*	rs231775	Italy	Case-control	Hospital	8	130	189
Salinas-Santander M [25]	2020	*CTLA4*	rs231775	Mexico	Case-control	Hospital	8	50	100

Variants described as RS code.

*Newcastle-Ottawa Scale (NOS) Score.

**Table 2:** Risk of evaluation bias of included studies using the Newcastle-Ottawa scale.

Study		Newcastle-Ottawa Domains								
		Selection				Comparability	Exposure			Total Score
First Author	Year	Adequate case definition	Representativeness of the cases	Selection of control	Definition of control	Control of important confusion factors	Ascertainment of exposure	Same method of ascertainment for cases and controls	Non-Response rate	
Betz RC	2008	1	1	1	0	2	1	1	1	8
Bhanusali D	2013	1	1	0	1	2	1	1	1	8
El-Zawahry B	2013	1	1	1	1	2	1	1	1	9
Kemp E	2006	1	1	0	1	2	1	1	1	8
Moravvej H	2018	1	1	1	1	2	1	1	1	9
Salinas-Santander M	2015	1	1	1	1	2	1	1	1	9
Shehata W	2020	1	1	1	1	2	1	1	1	9
Fan X	2010	1	1	0	1	2	1	1	1	8
Kalkan G	2013	1	1	0	1	2	1	1	1	8
Seleit I	2018	1	1	0	1	2	1	1	1	8
Tabatabaei-Panah P	2020	1	1	0	1	2	1	1	1	8
Miao Y	2014	1	1	0	1	2	1	1	1	8
Moravvej H	2018	1	1	1	1	2	1	1	1	9
Redler S	2012	1	1	1	1	2	1	1	1	9
Ismail N	2020	1	1	0	1	2	1	1	1	8
John K	2011	1	1	0	1	2	1	1	1	8
Megiorni F	2013	1	1	0	1	2	1	1	1	8
Salinas-Santander M	2020	1	1	0	1	2	1	1	1	8

**Table 3:** Case-control allelic and genotypic frequencies.

Study information					Type of alopecia areata				Cases					Controls				
Author	Year	Gene (RS)	Major Allele	Minor Allele	Patchy	Totalis	Universalis	AT/AU	Cases	Risk Alelle	WT	HT	HH	Controls	Risk Allele	WT	HT	HH
Betz RC	2008	PTPN22 (rs2476601)	C	T	196	.	.	239	435	126	320	104	11	628	132	506	112	10
Bhanusali D	2013	PTPN22 (rs2476601)	C	T	194	78	93	171	365	69	296	69	0	273	48	225	48	0
El-Zawahry B	2013	PTPN22 (rs2476601)	C	T	103	0	0	0	103	23	84	15	4	100	8	92	8	0
Kemp E	2006	PTPN22 (rs2476601)	C	T	107	.	.	84	196	41	155	41	0	507	86	425	79	3
Moravvej H	2018	PTPN22 (rs2476601)	C	T	69	0	0	0	69	24	50	14	5	69	23	55	5	9
Salinas-Santander M	2015	PTPN22 (rs2476601)	C	T	62	1	1	2	64	5	59	5	0	225	7	218	7	0
Shehata W	2020	PTPN22 (rs2476601)	C	T	.	.	.	.	60	36	32	.	.	30	6	25	.	.
Fan X	2010	FAS (rs1800682)	A	G	.	.	.	.	84	61	36	35	13	84	75	22	49	13
Kalkan G	2013	FAS (rs1800682)	A	G	118	0	0	0	118	81	37	81	0	118	91	40	65	13
Seleit I	2018	FAS (rs1800682)	A	G	30	15	15	30	60	65	9	37	14	40	31	13	23	4
Tabatabaei-Panah P	2020	FAS (rs1800682)	A	G	.	.	.	.	60	68	16	20	24	60	80	16	8	36
Kalkan G	2013	FASL (rs5030772)	A	G	118	0	0	0	118	42	78	38	2	118	43	40	65	13
Seleit I	2018	FASL (rs5030772)	A	G	30	15	15	30	60	50	18	34	8	40	22	24	10	6
Tabatabaei-Panah P	2020	FASL (rs5030772)	A	G	.	.	.	.	60	32	32	24	4	60	16	48	8	4
Miao Y	2014	IL2RA (rs3118470)	T	C	.	.	.	.	427	510	69	206	152	430	592	27	214	189
Moravvej H	2018	IL2RA (rs3118470)	T	C	69	0	0	0	69	44	39	16	14	69	16	59	5	5
Redler S	2012	IL2RA (rs3118470)	T	C	303	465	0	0	768	952	.	.	.	658	895	.	.	.
Ismail N	2020	CTLA4 (rs231775)	A	G	93	0	0	0	93	73	34	45	14	93	82	19	66	8
John K	2011	CTLA4 (rs231775)	A	G	.	.	.	.	1196	496	.	.	.	1280	462	.	.	.
Megiorni F	2013	CTLA4 (rs231775)	A	G	71	30	29	59	130	92	52	64	14	189	139	75	89	25
Salinas-Santander M	2020	CTLA4 (rs231775)	A	G	45	1	4	5	50	46	15	24	11	100	93	28	50	21

AT/AU: Alopecia totalis + Alopecia Universalis; WT: Wild Type genotype; HT: Heterozygous genotype; HH: Homozygous genotype.

### Assessment of risk of bias and quality of evidence

A study with an NOS score between 8 and 9 has high quality (Table 2). The GRADE score approach was used to grade the quality of associations such as *PTPN22* with AA. A moderate quality of evidence was observed for the *T* vs *C* allelic model and a high quality of evidence for the heterozygous and dominant co-dominant models (S3 File). The graphic and statistical evaluation of publication bias was not performed since the studies included were less than 10.

### Association of PTPN22 with the risk of developing alopecia areata

**Fig 2:**
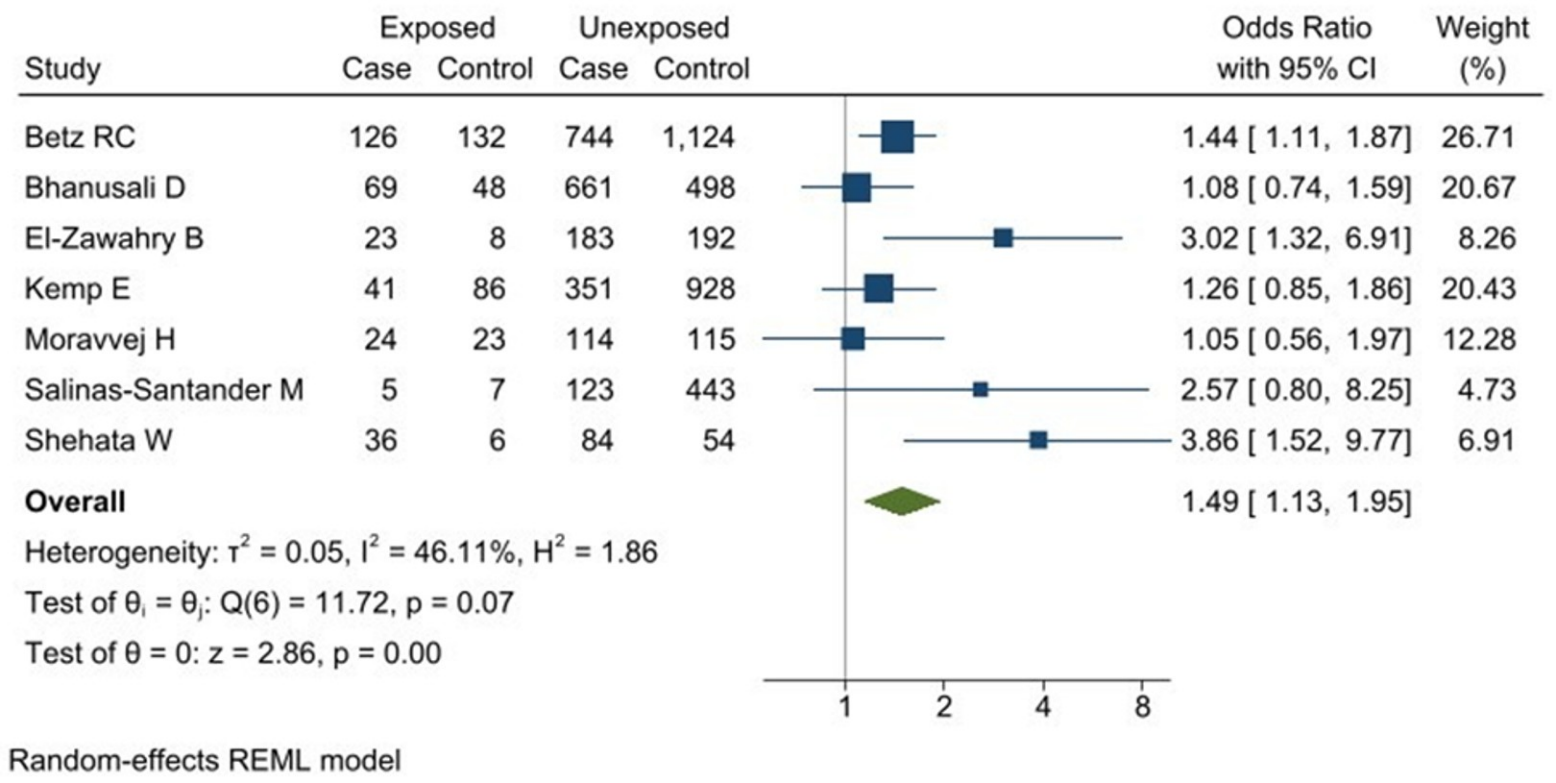
Association between rs2476601 polymorphism of *PTPN22* gene and the risk of alopecia areata. Allelic model T vs C. **Exposed group:** patients with allele T; **Unexposed group:** patients with allele *C*.

The combined analysis of 1292 cases and 1932 controls showed a statistically significant association between SNP rs2476601 polymorphism of *PTPN22* gene and the risk of developing AA. The presence of the *T* allele (rs2476601) was associated with the development of AA (OR1.49 [95% CI:1.13–1.95] *p* = 0.004) when using the allelic model (Fig 2). A subgroup analysis considering the NOS score was conducted to study the heterogeneity found in the model. A greater association was found between rs2476601 (*PTPN22*) and AA in the subgroup with a score of 9 (OR 2.22 [95% CI:1.16–4.24]) compared to the group with a score of 8 (OR 1.31 [95% CI:1.08–1.58]). The greatest heterogeneity was found in the group with a score of 9 (I^2^: 55.72%). A significant association was found (OR1.66 [95% CI:1.11–2.48]) in the community-based control groups (S4 and S5 Files) in the control group selection subgroup analysis.

**Fig 3:**
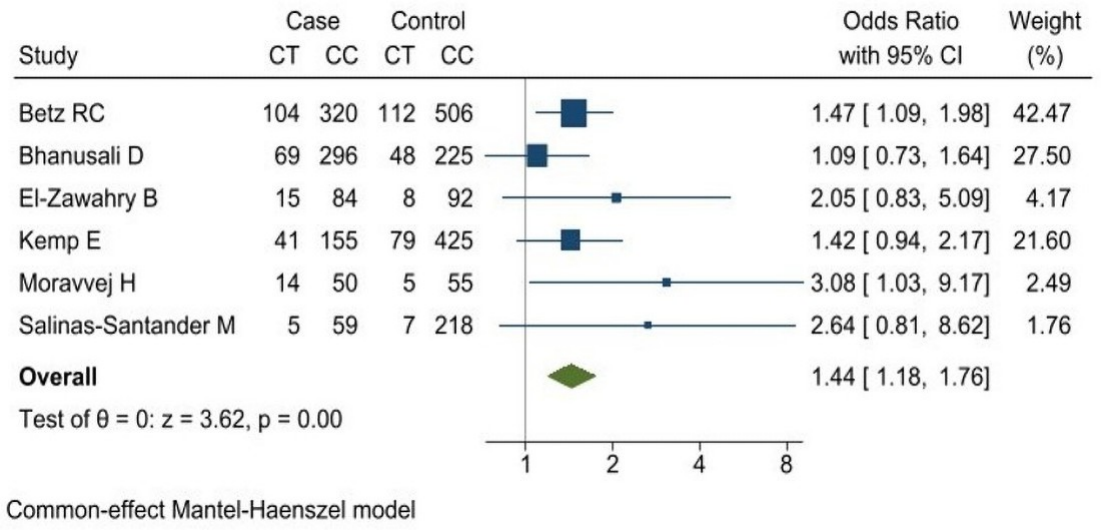
Association between rs2476601 polymorphism of *PTPN22* gene and risk of alopecia areata. Codominant model (*CT* vs *CC*).

**Fig 4:**
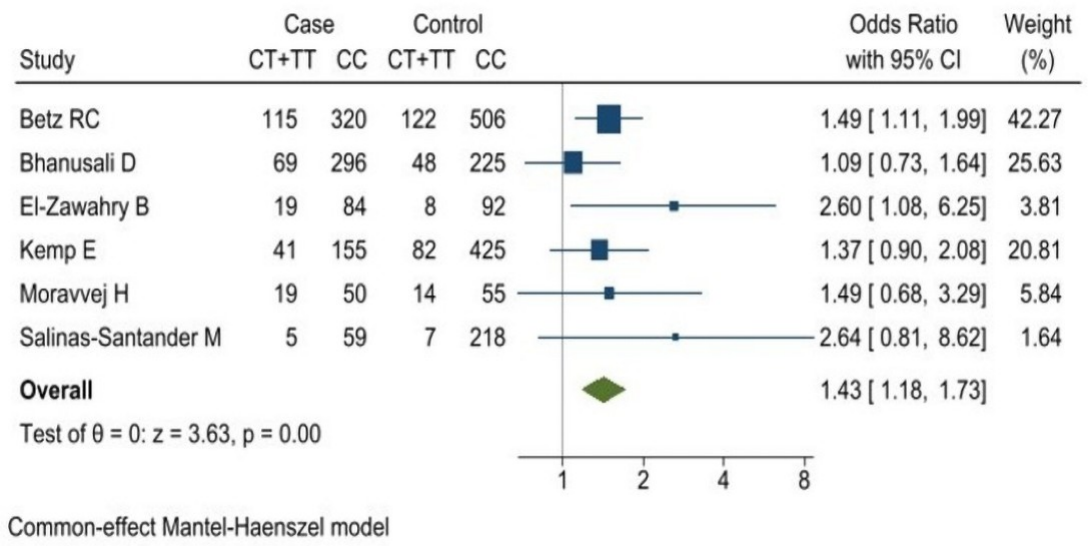
Association between rs2476601 polymorphism of *PTPN22* gene and risk of alopecia areata. Dominant model (*CT*+*TT* vs *CC*).

A combined analysis was performed for the homozygous codominant (*TT* vs *CC*), heterozygous codominant (*CT* vs *CC*), dominant (*TC* + *TT* vs *CC*) and recessive (*TT* vs *CC* + *CT*) models. The analysis for rs2476601 polymorphism of *PTPN22* gene evidenced that the risk of AA was 1.44-fold greater in subjects with the *CT* genotype compared with the *CC* genotype (OR 1.44 [95% CI: 1.18–1.76] *p* = 0.000) Fig 3. The dominant model (*TC* + *TT* vs *CC*) yielded an (OR of 1.43 [95% CI:1.18–1.73] *p* = 0.000) (Fig 4). The associations obtained for the homozygous codominant models and for the recessive model were not significant (Table 4).

**Table 4:** Association between genes *PTPN22*, *FAS/FASL* and *CTLA4* with alopecia areata.

Gen	Model	Allele/Genotype	Association			Meta-Analysis model	Heterogeneity	
			OR	IC-95%	p-value	Effect	I2%	*p*-value het
***PTPN22* (rs2476601)**	**Allelic**	T/C	1.49	1.13–1.95	**0.004** *	Random	46.11	0.068
	**Codominant**	CC	1	-	-	-	-	-
		CT	1.44	1.18–1.76	**0.000** *	Fixed	0	0.387
		TT	1.33	0.73–2.43	0.349	Fixed	13.25	0.434
	**Dominant**	CT + TT vs CC	1.43	1.18–1.73	**0.000** *	Fixed	0	0.464
	**Recessive**	TT vs CC +CT	1.2	0.66–2.18	0.549	Fixed	18.33	0.389
***FAS* (rs1800682)**	**Allelic**	G/A	0.9	0.60–1.34	0.598	Random	66.01	0.032
	**Codominant**	AA	1	-	-	-	-	-
		AG	-	-	-	-	75.47	0.007
		GG	-	-	-	-	83.02	0.01
	**Dominant**	AG + GG vs AA	1.03	0.55–1.96	0.917	Random	67.84	0.025
	**Recessive**	GG vs AA + AG	-	-	-	-	81,9	0,009
***FASL* (rs5030772)**	**Allelic**	G/A	1.57	0.91–2.71	0.108	Random	62.96	0.061
	**Codominant**	AA	1	-	-	-	-	-
		AG	-	-	-	-	93.61	0
		GG	-	-	-	-	82.85	0.004
	**Dominant**	AG +GG vs AA	-	-	-	-	94.1	0
	**Recessive**	GG vs AA + AG	0.524	0.16–1.70	0.282	Random	55.72	0.107
***CTLA4* (rs231775)**	**Allelic**	G/A	1.1	0.98–1.24	0.108	Fixed	36.67	0.243
	**Codominant**	AA	1	-	-	-	-	-
		AG	0.72	0.39–1.35	0.304	Random	64.7	0.056
		GG	0.89	0.54–1.49	0.663	Fixed	0	0.935
	**Dominant**	AG + GG vs AA	0.76	0.46–1.25	0.276	Random	49.9	0.136
	**Recessive**	GG vs AA + AG	1.08	0.68–1.69	0.751	Fixed	4.85	0.338

**p- value het**: chi square p value for heterogeneity.

Meta-analysis was not carried out when heterogeneity was greater than 75%.

*Statistically significant result.

### Association of *FAS* and *FASL* gene polymorphisms with the risk of developing alopecia areata

A meta-analysis of the allelic model for SNP rs1800682 FAS polymorphism, showed that the combined measure of the effect obtained from the allelic model (*G* vs *A*) was not significant (OR 0.9 [95% CI, 0.60–1.34] *p* = 0.598) (S5 File). To explore heterogeneity, a subgroup analysis was performed based on control group selection criteria. Subgroup analysis of the NOS score was not performed since all had a score of 8.

The association obtained for the dominant model was not significant (OR1.03 [95% CI: 0.55–1.96] *p* = 0.917). We decided not to include the homozygous codominant, heterozygous codominant and recessive models in our meta-analysis for a substantial heterogeneity was detected (Table 4).

The meta-analysis of the allelic model for SNP rs5030772 FASL polymorphism showed a not statistically significant (OR 1.57 [95% CI: 0.91–2.71] *p* = 0.108) (S5 and S6 Files) combined measure of the effect for the allelic approach (*G* vs *A*). When performing the subgroup analysis based on control group selection criteria, it was evidenced that the heterogeneity found was mainly given by the group of hospital-based controls. As for the results obtained for SNP rs1800682 FAS polymorphism, all the studies on rs5030772 FASL polymorphism had a NOS score of 8, thus, it was not possible to explain this parameter using a subgroup study analysis.

Regarding the combined analysis for the different genetic models, the association obtained for the recessive model was not statistically significant (OR 0.52 [95% CI: 0.16–1.70] *p* = 0.282). It was decided not to include the homozygous codominant, heterozygous codominant and dominant models in the meta-analysis, for a substantial heterogeneity was observed (Table 4).

### Association analysis of the CTLA4 gene with the risk of developing alopecia areata

When evaluating the relation between *CTLA4* rs231775 polymorphism and the risk of developing AA, the association obtained from the allelic model was not statistically significant (OR 1.1 [95% CI: 0.98–1.24] *p* = 0.108) (S6 and S7 Files).

The combined analysis for the homozygous codominant (*GG* vs *AA*), heterozygous codominant (*AG* vs *AA*), dominant (*AG* + *GG* vs *AA*) and recessive (*GG* vs *AA* + *AG*) models did not show any significant association (Table 4).

### Association analysis of the IL2RA gene with the risk of developing alopecia areata

To analyze the association of *IL2RA* rs3118470 polymorphism with the risk of developing AA, the combined analysis for the different genetic models: allelic (*T* vs *C*), homozygous codominant (*CC* vs *TT*), heterozygous codominant (*CT* vs *TT*), dominant (*CT* + *CC* vs *TT*) and recessive (*CC* vs *TT* + *CT* models, was proposed, but it was decided not to perform the meta-analysis due to the significant heterogeneity detected between genetic models.

## Discussion

### Genetic and pathophysiological bases of alopecia areata

AA is a multifactorial disease involving important immunological and genetic components in its pathogenesis, with a critical role played by both CD4^+^ and CD8^+^ T lymphocytes [1, 3]. Data from experimental studies have postulated CD8^+^ and NKG2D^+^ T lymphocytes as fundamental elements for the collapse of immune privilege of the hair follicle through the production of interferon-gamma, triggering an increase of IL-15 and a type I autoimmune reaction [26].

Given the polygenic nature of AA, genome-wide association studies (GWAS) have provided evidence on the involvement of genes related to both innate and adaptive immunity [5]. Variants associated with the development of AA were identified in at least 139 genes. The most relevant genes are associated with antigen presentation (*HLA-DRA*, *HLA-DQA1*, *HLA-DQA2*, *HLA-DQB2* and *HLA-A)*, with intracellular T lymphocyte signaling (*PTPN22*), encoding interleukins related to proliferation of T lymphocytes (*IL-21* and *IL-2*), interleukin receptors (*IL2RA*), inducers of T cell differentiation (*NOTCH-4*), costimulatory molecules (*CTLA4* and *ICOS*), the autoimmune response regulator gene (*AIR*), among other genes such as apoptosis and autophagy regulators (*ACOXL/BCL2L11*), as well as (*FAS/FASL*) [5, 10, 21, 27–29].

### Association of *PTPN22* with alopecia areata

The *PTPN22* gene encodes lymphoid tyrosine phosphatase (LYP) protein, which has been associated with several autoimmune diseases. This protein plays a role in the downregulation of the T cell receptor (TCR) and is essential in proliferation and maturation processes [30, 31]. LYP protein is potentiated by the C-terminal Src kinase (CSK) protein to generate the dephosphorylation of the T cell-specific tyrosine kinase (LCK) protein, and zeta chain of receptor associated kinase 70 (ZAP-70) protein, disrupting the TCR signaling cascade [30, 32].

Genetic variant rs2476601 polymorphism (involves the substitution of arginine for tryptophan, altering the P1 portion of LYP protein, whose function directly affects the interaction with the CSK protein altering the negative regulatory process made by the TCR, as there are fewer LYP-CSK complexes [33, 34].

In the present study, the meta-analysis data generated statistically significant results for the rs2476601 variant of the *PTPN22* gene and its association with the risk of developing AA in allelic models (OR1.49 [95% CI: 1.3–1.95]), heterozygous codominant (OR 1.44 [95% CI:1.19–1.76]) and dominant (OR1.43 [95% CI,1.18–1.73]), indicating that the presence of at least one T allele confers susceptibility to AA. These results are consistent with what was established by Kemp et al. [17] who conducted the first case-control study in a European population, finding the association of the *T* allele with severe forms of AA (OR1.89 [95% CI:1.17–3.05) in a group of 196 cases and 507 controls of English origin. Likewise, a systematic review by Lei et al. [35] determined the protective effect of the *C* allele (OR 0.77 [95% CI: 0.64–0.92]) in an allelic model using the data extracted from 5 primary studies. Similarly, they showed, by comparing the *CC* vs *CT* + *TT* genotypes, that carrying the CC genotype was associated with a decreased risk in the development of AA (OR 0.93 [95% C: 0.60–0.88]) [35].

The relationship with the variant (rs2476601) has been studied in various autoimmune diseases such as type-1 diabetes mellitus, finding that the *T* allele has been identified as a risk factor in North American and Italian populations [33]. In rheumatoid arthritis patients with positive rheumatoid factor, an OR of 1.5 [95% CI:1.1–1.9]), corresponding to the effect of the *T* allele, was found in an English population [36]. A systematic review carried out by Lea and Lee in systemic lupus erythematosus (SLE) patients had an OR of1.56 [95% CI: 1.33–1.82]) in the meta-analysis for the *T* allele, with primary studies including mainly European and Hispanic populations [37]. These findings showed that the *PTPN22* gene plays an important role in the regulation of immune homeostasis. Therefore, it is important to strengthen the knowledge on the frequency of the functional genetic variant rs2476601 and its role in the susceptibility to develop AA, considering aspects related to ethnicity, environmental factors, and geographic region of the population, given the heterogeneity found in studies reported in the literature. Large-sample studies are strongly recommended.

### Association of *CTLA4* with alopecia areata

The CTLA4 receptor is present in both CD4^+^ and CD8^+^ T cells. This protein is important for regulating immunity and maintaining immune tolerance [24, 38]. CTLA4 negatively regulates T lymphocytes by binding to protein B7, which is expressed by the antigen-presenting cell. Activation of the T lymphocyte involves the interaction of the TCR (T cell receptor) with the major histocompatibility complex loaded with the antigen on the surface of APC cells (antigen-presenting cells), but additionally requires co-stimulating signals that enhance the immune response or co-repressing signals that decrease this response (immune checkpoint). The co-stimulatory signal is given by the binding of the B7 protein (APC) to its ligand CD28 in the lymphocyte receptor, which increases the production of IL-2, proliferation, and the survival of the T lymphocyte [38, 39]. Co-repressing signals must be activated to maintain immunological homeostasis. CTLA 4, which is a co-inhibitory protein, competes with CD28 for binding to B7, when the CTLA4/B7 interaction is established, a signal that downregulates the T lymphocyte response is generated [11, 38, 39].

Alterations in the gene that encodes the CTLA4 receptor can trigger lymphocytic autoreactivity that has been postulated in AA pathogenesis, and CTLA4-mediated signaling plays an important role in preventing hair follicle immune privilege collapse [5, 24, 40]. The functional variant rs231775 (+49*G*/*A*) in the *CTLA4* gene produces a change from alanine to threonine in position 49 of exon 1 [41], which in turn generates an increase of the expression of CTLA4 in the cytoplasmic membrane, altering immunological homeostasis [42]. This variant, like the *PTPN22* gene, has been associated with susceptibility to the development of autoimmune diseases such as systemic lupus erythematosus, type-1 diabetes mellitus, and rheumatoid arthritis. A systematic review performed by Wang et al. suggests the use of rs231775 as a marker of susceptibility for the development of autoimmune diseases in Asian and Caucasian populations [43]. *CTLA4* upregulation has also been evidenced in neoplastic diseases such as pancreatic cancer [44].

The present study did not find a significant association between rs231775 polymorphism of the *CTLA4* gene with the development of AA. However, the studies showed heterogeneous results. John et al. [24] studied a great variety of genetic polymorphisms in *CTLA4* in Central Europe, but in the case of rs231775, the G allele was correlated with the development of AA (OR 1.26 [95% CI:1.12–1.41]), higher disease severity (OR1.43 [95% CI:1.24–1.64]) and early onset of the disease (OR1.39 [95% CI:1.20–1.61]) [24]. Megiorni et al. found no association between the rs231775 variant and the risk of developing AA, in an Italian population [12]. Ismail et al. found a protective effect on the *G* allele (OR: 0.44, 95% CI: 0.23–0.85) when compared to the homozygous form of the *A* allele (*GG* + *AG* vs *AA*), in an Egyptian male population [23] Finally, Salinas-Santander et al. study in a Mexican population, found no association between rs231775 and rs3087243 polymorphisms and the development of AA [25]. The later demonstrates that the rs231775 and rs3087243 variants of the CTLA4 gene play an important role in the pathophysiology of AA, however, they must be carefully interpreted according to the origin of the population.

### Association of *IL2RA* with alopecia areata

The *IL2RA* gene encodes the alpha chain of the IL-2 receptor (also known as CD25), which makes up one of the three receptor subunits and confers its high affinity to IL-2 on effector and regulatory T cells (CD4^+^ CD25^+^ FOXP3^+^) [45, 46]. These cells suppress autoreactive T lymphocytes and require IL-2 for their proper development and homeostasis, which is why alterations in this gene are correlated with immunodeficiency [47] or the development of autoimmune diseases such as type-1 diabetes mellitus, multiple sclerosis, systemic lupus erythematosus, rheumatoid arthritis and celiac disease [5, 48–50].

The rs3118470 (*T*>*C*) polymorphism of *IL2RA*, corresponds to an intronic variant located at the 5’ end of intron 1, the biological mechanism of the association of this variant with various diseases has not yet been determined. Separated at 3kb from this variant, another linkage disequilibrium polymorphism (rs706778) has been identified and has been associated with the development of type 1 diabetes mellitus [51].

In the present study, it was not possible to compute the combined measure of the effect of the included studies, given the substantial inter-study heterogeneity identified, which was mainly influenced by the clinical heterogeneity attributed to the fact that the studies were carried out in populations located in different geographical regions and a limited population in the included studies. The analysis by types of alopecia could not be performed since not all studies reported this feature.

Regarding individual results, Redler et al. study, in German and Belgium population, identified the *C* allele of the *IL2RA* rs706778 gene as a risk factor for developing AA (OR 1.3 [5% CI:1.12–1.51]), with a more severe form of the disease (OR 1.45 [95% CI,1.22–1.73]) in patients with a family history of AA (OR1.4 [95% CI:1.11–1.78]) [22]. Miao et al. study, in a Chinese population, found significant differences between frequencies of the *C* and *T* alleles; between cases and controls (*p* <0.0001). The study also evidenced that the allele and genotypic frequencies among the groups of severe and mild alopecia areata *p*-values results were not significant, as determined by the authors of the studies included in this systematic review (*p* = 0.289 and *p* = 0.137, respectively) [13]. Moravvej *et al*. considered the presence of the *C* allele (OR 3.56 [95% CI,1.89–6.71]) as a risk factor for developing AA [4].

### Association of *FAS/FASL* with alopecia areata

FAS/FASL pathway is critical for maintaining immunological homeostasis [52], due to the ability to induce programmed cell death in T cells, its role in the proliferation, activation and differentiation of T cells such as Th17 [6, 52–54]. In the AA context, it has been determined that variations in *FAS/FASL* genes could affect the apoptosis of T lymphocytes and natural killer cells involved in the pathogenesis of the disease [55]. Experimental studies have determined that FAS is expressed in hair follicles and FASL in perifollicular inflammatory infiltrate cells [56]. Therefore, as the FAS/FASL pathway is involved in the co-stimulation of both CD4^+^ and CD8^+^ during the early phases of immune response inducing apoptosis in follicular keratinocytes, it explains the absence of inflammatory infiltrates and resistance to AA development in mouse models with FAS/FASL deficiency [56, 57].

Functional rs1800682 polymorphism (−670 *A*>*G*) is found in the *FAS* gene promoter [58], specifically in the STAT1 (signal transducer and activator of transcription 1) binding site, a key element to initiate the process of transcriptional activation and expression of the *FAS* gene [59, 60]. Similarly, the rs5030772 variant (IVS2nt-124 A> G) is located in intron 2 and plays an important role in the transcription and expression of the *FASL* gene [58, 61].

The variants in the *FAS* and *FASL* genes did not show significant results in the present study. A meta-analysis was not performed for different genetic models since a high inter-study heterogeneity was identified. This could be explained by the small number of studies and sample size. However, rs1800682 has been reported to be associated with susceptibility to the development of AA. Kalkan *et al*. [10] identified allele *A* as a risk factor (OR 1.20 [95% CI: 0.82–1.75]) and *GG* genotype as a protective factor when comparing with *AG* and *AA* genotypes (OR 0.07 [95% CI: 0.00–0.4]) of the *FAS* rs1800682 variant. No significant findings were found for the rs5030772 variant of *FASL* gene [10]. In the study conducted by Fan *et al*. [19], a protective effect for the *GA* genotype when compared with the AA genotype (OR 0.43 [95% CI: 0.22–0.86]) was identified, and the homozygous form of this allele (*GG* genotype) showed a protective effect (OR 0.61 [95% CI, 0.23–0.86]). The effect of the 844*T*>*C* variant on the *FASL* gene was also assessed and no association with a combination with variants in the *FAS* gene was found [19].

Tabatabaei-Panah *et al*. [21] found no effect of *FAS* variant rs1800682 on the risk of developing AA, however, they did find an effect of both allele *A* (OR 2.36 [95% CI:1.21–4.59]) and genotype *AA* (OR 2.13 [95% CI:1.12–4.03]) of the rs5030772 variant of the *FASL* gene [21]. Finally, in a study carried out by Seleit *et al*. [20] in an Egyptian population, the presence of a *G* allele of the *FAS* variant rs1800682 conferred a risk effect (OR 1.75 [95% CI: 1.42–2.33]) as well as the homozygous form (*GG* genotype) of the same variant (OR 5.1 [95% CI: 1.25–20.48]), contrary to what was found by Kalkan *et al*. [10], who found a protective effect of the *GG* genotype in the Turkish population (OR 0.07 [95% CI: 0.00–0.4]) compared to *AG* + *AA* genotypes [10]. This heterogeneity may be influenced by geographical factors.

### Certainty of the evidence according to the GRADE approach

The results obtained by using the GRADEpro tool, made it possible to analyze the evidence for the allelic, codominant heterozygous and dominant genetic models, graded as moderate and high quality respectively regarding evidence on the *PTPN22* gene. This outcome was consistent with the individual evaluation of the studies using the NOS, in which individual studies obtained high scores. Although observational studies are known to have low quality of evidence, an adequate design and execution of primary studies allow an increase in the degree of certainty of the evidence.

### Limitations of the present study

The outcomes obtained in this study evidenced several limitations which must be considered for data interpretation. Regarding the *PTPN22* gene, it was not possible to analyze the subgroups by ethnicity, which usually allows a better interpretation of the data of multifactorial diseases such as AA. Furthermore, it was only possible to perform a meta-analysis for one variant per gene from the data obtained in the extraction phase, since not all studies analyzed the association between AA and the same gene polymorphisms. For that reason, we could not identify the contribution of each variant to the susceptibility to develop the disease.

Haplotype analysis may have provided more information regarding the role played by the variants in AA risk. On the other hand, the number of studies included for the combined analysis of each gene was limited, which reduces the statistical power of the estimates and allows us to identify that the estimates could be altered due to publication bias. Some of the analyses proposed could not be carried out since all the included studies did not consider the same variables, such as the different forms of AA.

The results obtained from the available studies for the genes included in this systematic review, evidence the need of further genetic association studies regarding genes that encode proteins that participate in the immunological pathway related to AA, in order to clarify the role that these variants have in the susceptibility to develop AA.

## Conclusion

This study confirms the association of the *PTPN22* gene rs2476601 variant with the risk of developing AA. This is evident in the increased risk that patients carrying the *T* allele have when compared to carrying the *C* allele. However, to perform more robust studies identifying the ethnic background of the population of origin, is required, so that the risk identified in the present study can be validated.

A statistical study in greater depth, on the effect of variants in the *CTLA4*, *FAS*/*FASL* and *IL2RA* genes, requires conducting a greater number of genetic association studies in order to calculate a combined effect measure.

## Supporting information

S1 File PRISMA 2009 checklist. (DOC)

S2 File Research strategy. (DOCX)

S3 File Quality of evidence assessment using GRADEpro tool. (DOCX)

S4 File Forest plot performed for the PTPN22 gene. PTPN22.
(DOCX)

S5 File Forest plot performed for the *FAS* gene. (DOCX)

S6 File Forest plot performed for the *FASL* gene. (DOCX)

S7 File Forest plot performed for *CTLA4* gene. (DOCX)

## References

[1] Pratt CH, King LE, Messenger AG, Christiano AM, Sundberg JP. Alopecia areata. Nat Rev Dis Primers. 2017;3: 17011. doi: 10.1038/nrdp.2017.11 28300084 PMC5573125

[2] Guo H, Cheng Y, Shapiro J, McElwee K. The role of lymphocytes in the development and treatment of alopecia areata. Expert Review of Clinical Immunology. 2015;11: 1335–1351. doi: 10.1586/1744666X.2015.1085306 26548356 PMC5148616

[3] Gilhar A, Etzioni A, Paus R. Alopecia Areata. N Engl J Med. 2012;366: 1515–25. doi: 10.1056/NEJMra1103442 22512484

[4] Moravvej H, Tabatabaei-Panah P-S, Abgoon R, Khaksar L, Sokhandan M, Tarshaei S, et al. Genetic variant association of PTPN22, CTLA4, IL2RA, as well as HLA frequencies in susceptibility to alopecia areata.: 15. 10.1080/08820139.2018.1480032 29979892

[5] Petukhova L, Duvic M, Hordinsky M, Norris D, Price V, Shimomura Y, et al. Genome-wide association study in alopecia areata implicates both innate and adaptive immunity. Nature. 2010;466: 113–117. doi: 10.1038/nature09114 20596022 PMC2921172

[6] Kennedy NJ, Kataoka T, Tschopp J, Budd RC. Caspase Activation Is Required for T Cell Proliferation. Journal of Experimental Medicine. 1999;190: 1891–1896. doi: 10.1084/jem.190.12.1891 10601363 PMC2195711

[7] Burn GL, Svensson L, Sanchez-Blanco C, Saini M, Cope AP. Why is PTPN22 a good candidate susceptibility gene for autoimmune disease? FEBS Letters. 2011;585: 3689–3698. doi: 10.1016/j.febslet.2011.04.032 21515266

[8] Abdulqader AMR, Mohammed AI, Rachid S. Polymorphisms in the cytotoxic T lymphocyte-associated protein-4 immune regulatory gene and their impact on inhibitor development in patients with hemophilia A. J Int Med Res. 2019;47: 4981–4992. doi: 10.1177/0300060519860329 31524022 PMC6833422

[9] Goudy K, Aydin D, Barzaghi F, Gambineri E, Vignoli M, Mannurita SC, et al. Human IL2RA null mutation mediates immunodeficiency with lymphoproliferation and autoimmunity. Clinical Immunology. 2013;146: 248–261. doi: 10.1016/j.clim.2013.01.004 23416241 PMC3594590

[10] Kalkan G, Ateş Ö, Karakuş N, Sezer S. Functional polymorphisms in cell death pathway genes FAS and FAS ligand and risk of alopecia areata. Arch Dermatol Res. 2013;305: 909–915. doi: 10.1007/s00403-013-1354-5 23591741

[11] Salinas-Santander M, Sánchez-Domínguez C, Cantú-Salinas C, Gonzalez-Cárdenas H, Cepeda-Nieto AC, Cerda-Flores RM, et al. Association between PTPN22 C1858T polymorphism and alopecia areata risk. Experimental and Therapeutic Medicine. 2015;10: 1953–1958. doi: 10.3892/etm.2015.2728 26640579 PMC4665763

[12] Megiorni F, Mora B, Maxia C, Gerardi M, Pizzuti A, Rossi A. Cytotoxic T-lymphocyte antigen 4 (CTLA4) +49AG and CT60 gene polymorphisms in Alopecia Areata: a case–control association study in the Italian population. Arch Dermatol Res. 2013;305: 665–670. doi: 10.1007/s00403-013-1348-3 23567921

[13] Miao Y, Kang Z, Xu F, Qi S, Sheng Y, Han Y, et al. Association Analysis of the IL2RA Gene with Alopecia Areata in a Chinese Population. Dermatology. 2013;227: 299–304. doi: 10.1159/000351555 24280705

[14] Betz RC, König K, Flaquer A, Redler S, Eigelshoven S, Kortüm A-K, et al. The R620W polymorphism in PTPN22 confers general susceptibility for the development of alopecia areata. Br J Dermatol. 2007;0: 071119222739011-??? doi: 10.1111/j.1365-2133.2007.08312.x 18028494

[15] Bhanusali DG, Sachdev A, Olson MA, Gerlach JA, Sinha AA. PTPN22 profile indicates a novel risk group in Alopecia areata. Human Immunology. 2014;75: 81–87. doi: 10.1016/j.humimm.2013.09.003 24055692

[16] El-Zawahry BM, Azzam OA, Zaki NS, Abdel-Raheem HM, Bassiouny DA, Khorshied MM. PTPN22 gene polymorphism in Egyptian alopecia areata patients and its impact on response to diphencyprone immunotherapy. Gene. 2013;523: 147–151. doi: 10.1016/j.gene.2013.03.070 23570882

[17] Kemp EH, McDonagh AJG, Wengraf DA, Messenger AG, Gawkrodger DJ, Cork MJ, et al. The Non-Synonymous C1858T Substitution in the PTPN22 Gene is Associated with Susceptibility to the Severe Forms of Alopecia Areata. Human Immunology. 2006;67: 535–539. doi: 10.1016/j.humimm.2006.04.006 16829308

[18] Shehata WA, Maraee A, Kamal H, Tayel N, Azmy R. Protein tyrosine phosphatase nonreceptor type 22 gene polymorphism in alopecia areata: Does it have an association with disease severity? J Cosmet Dermatol. 2020;19: 3138–3144. doi: 10.1111/jocd.13412 32281251

[19] Fan X, Shangguan L, Li M, Li CY, Liu B. Functional polymorphisms of the FAS / FASLG genes are associated with risk of alopecia areata in a Chinese population: a case-control analysis: FAS / FASLG gene polymorphisms and alopecia areata risk. British Journal of Dermatology. 2010;163: 340–344. doi: 10.1111/j.1365-2133.2010.09808.x 20394629

[20] Seleit I, Bakry OA, Gayed EAE, Gawad AED. Polymorphism of FAS and FAS Ligand Genes in Alopecia Areata: A Case–control Study in Egyptian Population.: 14. 10.4103/ijd.IJD_286_17 PMC5996631 29937558

[21] Tabatabaei-Panah P-S, Moravvej H, Arian S, Fereidonpour I, Behravesh N, Atoon A, et al. Overlapping and Distinct FAS/FASLG Gene Polymorphisms in Alopecia Areata in an Iranian Population. IMMUNOLOGICAL INVESTIGATIONS.: 12. 10.1080/08820139.2019.1688827 31741398

[22] Redler S, Albert F, Brockschmidt FF, Herold C, Hanneken S, Eigelshoven S, et al. Investigation of selected cytokine genes suggests that IL2RA and the TNF / LTA locus are risk factors for severe alopecia areata. British Journal of Dermatology. 2012;167: 1360–1365. doi: 10.1111/bjd.12004 22897480

[23] Ismail NA, Toraih EA, Ameen HM, Gomaa AHA, Marie RE-SM. Association of Rs231775 Genetic Variant of Cytotoxic T-lymphocyte Associated Protein 4 with Alopecia Areata Disease in Males: A Case–Control Study. Immunological Investigations. 2020; 1–10. doi: 10.1080/08820139.2020.1796700 32731768

[24] John KK-G, Brockschmidt FF, Redler S, Herold C, Hanneken S, Eigelshoven S, et al. Genetic Variants in CTLA4 Are Strongly Associated with Alopecia Areata. Journal of Investigative Dermatology. 2011;131: 1169–1172. doi: 10.1038/jid.2010.427 21346773

[25] Salinas-Santander MA, Cantu-Salinas CS, Ocampo-Candiani J, Suarez-Valencia V de J, Ramirez-Guerrero JG, Sanchez-Dominguez CN. CTLA4 +49AG (rs231775) and CT60 (rs3087243) gene variants are not associated with alopecia areata in a Mexican population from Monterrey Mexico. Anais Brasileiros de Dermatologia. 2020;95: 283–288. doi: 10.1016/j.abd.2020.03.001 32278632 PMC7253907

[26] Xing L, Dai Z, Jabbari A, Cerise JE, Higgins CA, Gong W, et al. Alopecia areata is driven by cytotoxic T lymphocytes and is reversed by JAK inhibition. Nat Med. 2014;20: 1043–1049. doi: 10.1038/nm.3645 25129481 PMC4362521

[27] Petukhova L, Christiano AM. Functional Interpretation of Genome-Wide Association Study Evidence in Alopecia Areata. Journal of Investigative Dermatology. 2016;136: 314–317. doi: 10.1038/JID.2015.402 26763452 PMC4870380

[28] Betz RC, Petukhova L, Ripke S, Huang H, Menelaou A, Redler S, et al. Genome-wide meta-analysis in alopecia areata resolves HLA associations and reveals two new susceptibility loci. Nat Commun. 2015;6: 5966. doi: 10.1038/ncomms6966 25608926 PMC4451186

[29] Jabbari A, Petukhova L, Cabral RM, Clynes R, Christiano AM. Genetic Basis of Alopecia Areata. Dermatologic Clinics. 2013;31: 109–117. doi: 10.1016/j.det.2012.08.014 23159180 PMC4362526

[30] Wu J, Katrekar A, Honigberg LA, Smith AM, Conn MT, Tang J, et al. Identification of Substrates of Human Protein-tyrosine Phosphatase PTPN22. J Biol Chem. 2006;281: 11002–11010. doi: 10.1074/jbc.M600498200 16461343

[31] Mustelin T. Protein tyrosine phosphatases in T cell physiology. Molecular Immunology. 2004;41: 687–700. doi: 10.1016/j.molimm.2004.04.015 15220004

[32] Gianchecchi E, Palombi M, Fierabracci A. The putative role of the C1858T polymorphism of protein tyrosine phosphatase PTPN22 gene in autoimmunity. Autoimmunity Reviews. 2013;12: 717–725. doi: 10.1016/j.autrev.2012.12.003 23261816

[33] Bottini N, Musumeci L, Alonso A, Rahmouni S, Nika K, Rostamkhani M, et al. A functional variant of lymphoid tyrosine phosphatase is associated with type I diabetes. Nat Genet. 2004;36: 337–338. doi: 10.1038/ng1323 15004560

[34] Bottini N, Vang T, Cucca F, Mustelin T. Role of PTPN22 in type 1 diabetes and other autoimmune diseases. Seminars in Immunology. 2006;18: 207–213. doi: 10.1016/j.smim.2006.03.008 16697661

[35] Lei Z-X, Chen W-J, Liang J-Q, Wang Y-J, Jin L, Xu C, et al. The association between rs2476601 polymorphism in PTPN22 gene and risk of alopecia areata: A meta-analysis of case–control studies. Medicine. 2019;98: e15448. doi: 10.1097/MD.0000000000015448 31096440 PMC6531179

[36] Harrison P, Pointon JJ, Farrar C, Brown MA, Wordsworth BP. Effects of PTPN22 C1858T polymorphism on susceptibility and clinical characteristics of British Caucasian rheumatoid arthritis patients. Rheumatology. 2006;45: 1009–1011. doi: 10.1093/rheumatology/kei250 16490755

[37] Lea W, Lee Y. The association between the PTPN22 C1858T polymorphism and systemic lupus erythematosus: a meta-analysis update. Lupus. 2011;20: 51–57. doi: 10.1177/0961203310381774 21078766

[38] Rowshanravan B, Halliday N, Sansom DM. CTLA-4: a moving target in immunotherapy. Blood. 2018;131: 58–67. doi: 10.1182/blood-2017-06-741033 29118008 PMC6317697

[39] Buchbinder EI, Desai A. CTLA-4 and PD-1 Pathways: Similarities, Differences, and Implications of Their Inhibition. American Journal of Clinical Oncology. 2016;39: 98–106. doi: 10.1097/COC.0000000000000239 26558876 PMC4892769

[40] Paus R, Bertolini M. The Role of Hair Follicle Immune Privilege Collapse in Alopecia Areata: Status and Perspectives. Journal of Investigative Dermatology Symposium Proceedings. 2013;16: S25–S27. doi: 10.1038/jidsymp.2013.7 24326544

[41] Lang C, Chen L, Li S. Cytotoxic T-Lymphocyte Antigen-4 +49G/A Polymorphism and Susceptibility to Pancreatic Cancer. DNA and Cell Biology. 2012;31: 683–687. doi: 10.1089/dna.2011.1417 22011251

[42] Ligers A, Teleshova N, Masterman T, Huang W-X, Hillert J. CTLA-4 gene expression is influenced by promoter and exon 1 polymorphisms. Genes Immun. 2001;2: 145–152. doi: 10.1038/sj.gene.6363752 11426323

[43] Wang K, Zhu Q, Lu Y, Lu H, Zhang F, Wang X, et al. CTLA-4 +49 G/A Polymorphism Confers Autoimmune Disease Risk: An Updated Meta-Analysis. Genetic Testing and Molecular Biomarkers. 2017;21: 222–227. doi: 10.1089/gtmb.2016.0335 28384040

[44] Loos M, Giese NA, Kleeff J, Giese T, Gaida MM, Bergmann F, et al. Clinical significance and regulation of the costimulatory molecule B7-H1 in pancreatic cancer. Cancer Letters. 2008;268: 98–109. doi: 10.1016/j.canlet.2008.03.056 18486325

[45] Kawasaki E, Awata T, Ikegami H, Kobayashi T, Maruyama T, Nakanishi K, et al. Genetic Association between the Interleukin-2 Receptor-α Gene and Mode of Onset of Type 1 Diabetes in the Japanese Population. The Journal of Clinical Endocrinology & Metabolism. 2009;94: 947–952. doi: 10.1210/jc.2008-1596 19106270

[46] Kim HP, Imbert J, Leonard WJ. Both integrated and differential regulation of components of the IL-2/IL-2 receptor system. Cytokine & Growth Factor Reviews. 2006;17: 349–366. doi: 10.1016/j.cytogfr.2006.07.003 16911870

[47] Fichna M, Żurawek M, Bratland E, Husebye ES, Kasperlik-Załuska A, Czarnocka B, et al. Interleukin-2 and subunit alpha of its soluble receptor in autoimmune Addison’s disease–An association study and expression analysis. Autoimmunity. 2015;48: 100–107. doi: 10.3109/08916934.2014.976628 25347332

[48] Qu H-Q, Bradfield JP, Bélisle A, Grant SFA, Hakonarson H, Polychronakos C. The type I diabetes association of the IL2RA locus. Genes Immun. 2009;10: S42–S48. doi: 10.1038/gene.2009.90 19956099 PMC2805446

[49] Carr EJ, Clatworthy MR, Lowe CE, Todd JA, Wong A, Vyse TJ, et al. Contrasting genetic association of IL2RAwith SLE and ANCA–associated vasculitis. BMC Med Genet. 2009;10: 22. doi: 10.1186/1471-2350-10-22 19265545 PMC2662820

[50] Cavanillas ML, Alcina A, Núñez C, de las Heras V, Fernández-Arquero M, Bartolomé M, et al. Polymorphisms in the IL2, IL2RA and IL2RB genes in multiple sclerosis risk. Eur J Hum Genet. 2010;18: 794–799. doi: 10.1038/ejhg.2010.15 20179739 PMC2987360

[51] Qu H-Q, Montpetit A, Ge B, Hudson TJ, Polychronakos C. Toward Further Mapping of the Association Between the IL2RA Locus and Type 1 Diabetes. Diabetes. 2007;56: 1174–1176. doi: 10.2337/db06-1555 17395754

[52] Shen Y, Song Z, Lu X, Ma Z, Lu C, Zhang B, et al. Fas signaling-mediated TH9 cell differentiation favors bowel inflammation and antitumor functions. Nat Commun. 2019;10: 2924. doi: 10.1038/s41467-019-10889-4 31266950 PMC6606754

[53] Paulsen M, Janssen O. Pro- and anti-apoptotic CD95 signaling in T cells. Cell Commun Signal. 2011;9: 7. doi: 10.1186/1478-811X-9-7 21477291 PMC3090738

[54] Meyer zu Horste G, Przybylski D, Schramm MA, Wang C, Schnell A, Lee Y, et al. Fas Promotes T Helper 17 Cell Differentiation and Inhibits T Helper 1 Cell Development by Binding and Sequestering Transcription Factor STAT1. Immunity. 2018;48: 556–569.e7. doi: 10.1016/j.immuni.2018.03.008 29562202

[55] Yoo Y-G, Lee M-O. Hepatitis B Virus X Protein Induces Expression of Fas Ligand Gene through Enhancing Transcriptional Activity of Early Growth Response Factor. J Biol Chem. 2004;279: 36242–36249. doi: 10.1074/jbc.M401290200 15173177

[56] Freyschmidt-Paul P, McElwee KJ, Botchkarev V, Kissling S, Wenzel E, Sundberg JP, et al. Fas-Deficient C3.MRL-Tnfrsf6lpr Mice and Fas Ligand-De¢cient C3H/HeJ-Tnfsf6gld Mice Are Relatively Resistant to the Induction of Alopecia Areata by Grafting of Alopecia Areata-Affected Skin from C3H/HeJ Mice. 2003;8: 5. 10.1046/j.1523-1747.2003.12182.x 12895005

[57] Suzuki I, Fink PJ. The dual functions of Fas ligand in the regulation of peripheral CD8+ and CD4+ T cells. Proceedings of the National Academy of Sciences. 2000;97: 1707–1712. doi: 10.1073/pnas.97.4.1707 10677522 PMC26500

[58] Hashemi M, Fazaeli A, Ghavami S, Eskandari-Nasab E, Arbabi F, Mashhadi MA, et al. Functional Polymorphisms of FAS and FASL Gene and Risk of Breast Cancer–Pilot Study of 134 Cases. Lau KM, editor. PLoS ONE. 2013;8: e53075. doi: 10.1371/journal.pone.0053075 23326385 PMC3543397

[59] Huang QR, Morris D, Manolios N. Identification and characterisation of polymorphisms in the promoter region of the human Apo-1/Fas (CD95) gene. Molecular Immunology. 1997;34: 577–582. doi: 10.1016/s0161-5890(97)00081-3 9393960

[60] Sibley K, Rollinson S, Allan JM, Smith AG, Law GR, Roddam PL, et al. Functional FAS Promoter Polymorphisms Are Associated with Increased Risk of Acute Myeloid Leukemia.: 5. 12907599

[61] Takahashi T, Tanaka M, Inazawa J, Abe T, Suda T, Nagata S. Human Fas ligand: gene structure, chromosomal location and species specificity. Int Immunol. 1994;6: 1567–1574. doi: 10.1093/intimm/6.10.1567 7826947

