# Supplementary material for: Effect of PTPN22, FAS/FASL, IL2RA and CTLA4 genetic polymorphisms on the risk of developing alopecia areata: A systematic review of the literature and meta-analysis
Source: PLoS One. 2021 Nov 4;16(11):e0258499. doi: 10.1371/journal.pone.0258499 (PMC8568157; doi:10.1371/journal.pone.0258499)
Supplement: S2 File — (DOCX) [file pone.0258499.s002.docx]

**Research strategy**

(Search date for all databases until June 2020)

**Medline database**

***FAS* gene and alopecia areata**

 ((((((((((((Population[MeSH Terms]) OR (((populations) OR school age population) OR school age populations))) OR population))) OR ((((((((Adult[MeSH Terms]) OR Aged[MeSH Terms]) OR (Aged, 80[MeSH Terms] AND over[MeSH Terms])) OR Frail Elderly[MeSH Terms]) OR Middle Aged[MeSH Terms]) OR Young Adult[MeSH Terms])) OR (((((((((((adult, young) OR adults, young) OR young adults) OR middle age) OR oldest old) OR nonagenarians) OR nonagenarian) OR octogenarian) OR octogenarians) OR centenarian) OR centenarians))) OR ((Adolescent[MeSH Terms]) OR ((((((((adolescents) OR adolescence) OR adolescent) OR teens) OR teenagers) OR teenager) OR youth) OR youths))) OR ((((((((((children) OR preschool child) OR children, preschool) OR preschool children) OR toddler) OR Kid) OR kids) OR childhood) OR pediatric) OR infants)))) AND (((((fas receptor[MeSH Terms]) OR fas receptors[MeSH Terms]) OR FAS protein, human[Supplementary Concept])) OR ((((((((((((((((((((((((((((((((((receptor, fas) OR fas antigen) OR fas antigens) OR apo-1 antigen) OR apo 1 antigen) OR cd95 antigen) OR tnfrsf6 receptor) OR receptor, tnfrsf6) OR antigens, cd95) OR fas receptors) OR tumor necrosis factor receptor superfamily member 6) OR fas cell surface death receptor) OR cd95 antigens) OR cd 95 antigen, human) OR tumor necrosis factor receptor superfamily member 6 protein, human) OR fas1 protein, human) OR Fas (tnf receptor superfamily, member 6) , human) OR tnfrsf6 protein, human) OR APO-1) OR apo-1 antigen) OR apo-1 cell surface antigen) OR apoptosis antigen 1) OR apoptosis-mediating surface antigen fas) OR APT1) OR cd95 antigen) OR CD95) OR Fas (tnf receptor superfamily, member 6)) OR fas ama) OR fas antigen) OR FAS1) OR fas receptor) OR TNFRSF6) OR tnr6_human) OR tumor necrosis factor receptor superfamily member 6))) OR (((((Genetic Variation[MeSH Terms]) OR Polymorphism, Genetic[MeSH Terms]) OR Polymorphism, Single Nucleotide[MeSH Terms])) AND ((((((((((((((((((((genetic variations) OR variations, genetic) OR variation, genetic) OR diversity, genetic) OR diversities, genetic) OR genetic diversities) OR genetic diversity) OR polymorphisms, genetic) OR genetic polymorphisms) OR genetic polymorphism) OR polymorphism (genetics)) OR polymorphisms (genetics)) OR nucleotide polymorphism, single) OR nucleotide polymorphisms, single) OR polymorphisms, single nucleotide) OR single nucleotide polymorphisms) OR snps) OR single nucleotide polymorphism) OR genetic variant) OR genetic variants)) AND ((((alopecia areata[MeSH Terms]) OR alopecia circumscripta) OR alopecia totalis) OR alopecia universalis)

***FASL* gene and alopecia areata**

(((((((((((((Population[MeSH Terms]) OR (((Populations) OR School Age Population) OR School Age Populations))) OR Population))) OR ((((((((Adult[MeSH Terms]) OR Aged[MeSH Terms]) OR (Aged, 80[MeSH Terms] AND over[MeSH Terms])) OR Frail Elderly[MeSH Terms]) OR Middle Aged[MeSH Terms]) OR Young Adult[MeSH Terms])) OR (((((((((((Adult, young) OR Adults, young) OR Young adults) OR Middle Age) OR Oldest Old) OR Nonagenarians) OR Nonagenarian) OR Octogenarian) OR Octogenarians) OR Centenarian) OR Centenarians))) OR ((Adolescent[MeSH Terms]) OR ((((((((Adolescents) OR Adolescence) OR Adolescent) OR Teens) OR Teenagers) OR Teenager) OR Youth) OR Youths))) OR ((((((((((Children) OR Preschool Child) OR Children, Preschool) OR Preschool Children) OR Toddler) OR Kid) OR Kids) OR Childhood) OR Pediatric) OR Infants)))) AND ((((Fas Ligand Protein[MeSH Terms]) OR FASLG protein, human [Supplementary Concept])) AND ((((((((((((((((((CD95L) OR Fas Ligand (FasL)) OR FasL Protein) OR TNF Superfamily, Member 6) OR CD178 antigen) OR Antigen CD178) OR CD178 Antigens) OR CD95 Antigen Ligand) OR CD95 Ligand) OR ALPS1B) OR APTLG1) OR APTL) OR CD95-L) OR CD95L) OR CD178) OR FASL) OR TNFSF6) OR TNLG1A))) AND (((((Genetic Variation[MeSH Terms]) OR Polymorphism, Genetic[MeSH Terms]) OR Polymorphism, Single Nucleotide[MeSH Terms])) AND ((((((((((((((((((((Genetic Variations) OR Variations, Genetic) OR Variation, Genetic) OR Diversity, Genetic) OR Diversities, Genetic) OR Genetic Diversities) OR Genetic Diversity) OR Polymorphisms, Genetic) OR Genetic Polymorphisms) OR Genetic Polymorphism) OR Polymorphism (Genetics)) OR Polymorphisms (Genetics)) OR Nucleotide Polymorphism, Single) OR Nucleotide Polymorphisms, Single) OR Polymorphisms, Single Nucleotide) OR Single Nucleotide Polymorphisms) OR SNPs) OR Single Nucleotide Polymorphism) OR Genetic Variant) OR Genetic Variants))) AND ((((alopecia areata[MeSH Terms]) OR Alopecia Circumscripta) OR Alopecia totalis) OR Alopecia universalis))

***PTPN22 gene and alopecia areata***

((((((((((((Population[MeSH Terms]) OR (((Populations) OR School Age Population) OR School Age Populations))) OR Population))) OR ((((((((Adult[MeSH Terms]) OR Aged[MeSH Terms]) OR (Aged, 80[MeSH Terms] AND over[MeSH Terms])) OR Frail Elderly[MeSH Terms]) OR Middle Aged[MeSH Terms]) OR Young Adult[MeSH Terms])) OR (((((((((((Adult, young) OR Adults, young) OR Young adults) OR Middle Age) OR Oldest Old) OR Nonagenarians) OR Nonagenarian) OR Octogenarian) OR Octogenarians) OR Centenarian) OR Centenarians))) OR ((Adolescent[MeSH Terms]) OR ((((((((Adolescents) OR Adolescence) OR Adolescent) OR Teens) OR Teenagers) OR Teenager) OR Youth) OR Youths))) OR ((((((((((Children) OR Preschool Child) OR Children, Preschool) OR Preschool Children) OR Toddler) OR Kid) OR Kids) OR Childhood) OR Pediatric) OR Infants)))) AND ((((Protein Tyrosine Phosphatase, Non-Receptor Type 22[MeSH Terms]) OR PTPN22 protein, human [Supplementary Concept])) OR ((((((((((((((((((((((((((((((((Protein Tyrosine Phosphatase, Non Receptor Type 22) OR PTPase Lyp) OR Lymphoid Phosphatase) OR Tyrosine Protein Phosphatase, Non-Receptor Type 22) OR Tyrosine Protein Phosphatase, Non Receptor Type 22) OR PTPN-22 Protein) OR PTPN 22 Protein) OR Protein-Tyrosine Phosphatase Lyp) OR Protein Tyrosine Phosphatase Lyp) OR Lyp1 PTPase) OR PTPase, Lyp1) OR Lyp2 PTPase) OR PTPase, Lyp2) OR protein tyrosine phosphatase, non-receptor type 8, human) OR PTPN8 protein, human) OR protein tyrosine phosphatase, non-receptor type 22 (lymphoid), human) OR protein tyrosine phosphatase-22, human) OR Lyp1 protein, human) OR Lyp2 protein, human) OR hematopoietic cell protein-tyrosine phosphatase 70Z-PEP) OR lymphoid phosphatase) OR lymphoid-specific protein tyrosine phosphatase) OR LYP) OR LYP1) OR LYP2) OR PEP) OR PEST-domain phosphatase) OR tyrosine-protein phosphatase non-receptor type 22) OR PTPN8) OR PTN22_HUMAN) OR protein tyrosine phosphatase, non-receptor type 22 (lymphoid)) OR protein tyrosine phosphatase, non-receptor type 8))) AND (((((Genetic Variation[MeSH Terms]) OR Polymorphism, Genetic[MeSH Terms]) OR Polymorphism, Single Nucleotide[MeSH Terms])) AND ((((((((((((((((((((Genetic Variations) OR Variations, Genetic) OR Variation, Genetic) OR Diversity, Genetic) OR Diversities, Genetic) OR Genetic Diversities) OR Genetic Diversity) OR Polymorphisms, Genetic) OR Genetic Polymorphisms) OR Genetic Polymorphism) OR Polymorphism (Genetics)) OR Polymorphisms (Genetics)) OR Nucleotide Polymorphism, Single) OR Nucleotide Polymorphisms, Single) OR Polymorphisms, Single Nucleotide) OR Single Nucleotide Polymorphisms) OR SNPs) OR Single Nucleotide Polymorphism) OR Genetic Variant) OR Genetic Variants))) AND ((((alopecia areata[MeSH Terms]) OR Alopecia Circumscripta) OR Alopecia totalis) OR Alopecia universalis)

***CTLA4* gene and alopecia areata**

((((((((((((Population[MeSH Terms]) OR (((Populations) OR School Age Population) OR School Age Populations))) OR Population))) OR ((((((((Adult[MeSH Terms]) OR Aged[MeSH Terms]) OR (Aged, 80[MeSH Terms] AND over[MeSH Terms])) OR Frail Elderly[MeSH Terms]) OR Middle Aged[MeSH Terms]) OR Young Adult[MeSH Terms])) OR (((((((((((Adult, young) OR Adults, young) OR Young adults) OR Middle Age) OR Oldest Old) OR Nonagenarians) OR Nonagenarian) OR Octogenarian) OR Octogenarians) OR Centenarian) OR Centenarians))) OR ((Adolescent[MeSH Terms]) OR ((((((((Adolescents) OR Adolescence) OR Adolescent) OR Teens) OR Teenagers) OR Teenager) OR Youth) OR Youths))) OR ((((((((((Children) OR Preschool Child) OR Children, Preschool) OR Preschool Children) OR Toddler) OR Kid) OR Kids) OR Childhood) OR Pediatric) OR Infants)))) AND ((((CTLA-4 Antigen[MeSH Terms]) OR CTLA4 protein, human [Supplementary Concept])) OR (((((((((((((((((((((Antigen, CTLA-4) OR CTLA 4 Antigen) OR Antigens, CD152) OR CD152 Antigens) OR Cytotoxic T-Lymphocyte-Associated Antigen 4) OR Cytotoxic T Lymphocyte Associated Antigen 4) OR Cytotoxic T-Lymphocyte Antigen 4) OR Cytotoxic T Lymphocyte Antigen 4) OR CD152 Antigen) OR Antigen, CD152) OR cytotoxic T-lymphocyte-associated protein 4, human) OR CTLA-4 protein, human) OR CD152 antigen, human) OR ALPS5) OR CD) OR CD152) OR CELIAC3) OR CTLA-4) OR GRD4) OR GSE) OR IDDM12))) AND (((((Genetic Variation[MeSH Terms]) OR Polymorphism, Genetic[MeSH Terms]) OR Polymorphism, Single Nucleotide[MeSH Terms])) AND ((((((((((((((((((((Genetic Variations) OR Variations, Genetic) OR Variation, Genetic) OR Diversity, Genetic) OR Diversities, Genetic) OR Genetic Diversities) OR Genetic Diversity) OR Polymorphisms, Genetic) OR Genetic Polymorphisms) OR Genetic Polymorphism) OR Polymorphism (Genetics)) OR Polymorphisms (Genetics)) OR Nucleotide Polymorphism, Single) OR Nucleotide Polymorphisms, Single) OR Polymorphisms, Single Nucleotide) OR Single Nucleotide Polymorphisms) OR SNPs) OR Single Nucleotide Polymorphism) OR Genetic Variant) OR Genetic Variants))) AND ((((alopecia areata[MeSH Terms]) OR Alopecia Circumscripta) OR Alopecia totalis) OR Alopecia universalis)

***IL2RA* gene and alopecia areata**

((((((((((((Population[MeSH Terms]) OR (((populations) OR school age population) OR school age populations))) OR population))) OR ((((((((Adult[MeSH Terms]) OR Aged[MeSH Terms]) OR (Aged, 80[MeSH Terms] AND over[MeSH Terms])) OR Frail Elderly[MeSH Terms]) OR Middle Aged[MeSH Terms]) OR Young Adult[MeSH Terms])) OR (((((((((((adult, young) OR adults, young) OR young adults) OR middle age) OR oldest old) OR nonagenarians) OR nonagenarian) OR octogenarian) OR octogenarians) OR centenarian) OR centenarians))) OR ((Adolescent[MeSH Terms]) OR ((((((((adolescents) OR adolescence) OR adolescent) OR teens) OR teenagers) OR teenager) OR youth) OR youths))) OR ((((((((((children) OR preschool child) OR children, preschool) OR preschool children) OR toddler) OR Kid) OR kids) OR childhood) OR pediatric) OR infants)))) AND ((((Interleukin-2 Receptor alpha Subunit[MeSH Terms]) OR IL2RA protein, human[Supplementary Concept])) AND (((((((((((((((((((((((((((interleukin 2 receptor, alpha protein, human) OR cd25 antigen, human) OR interleukin 2 receptor alpha subunit) OR antigens, cd25) OR cd25 antigens) OR tac p55 peptide) OR interleukin-2 receptor alpha) OR interleukin 2 receptor alpha) OR receptor alpha, interleukin-2) OR interleukin-2 receptor alpha chain) OR interleukin 2 receptor alpha chain) OR interleukin-2 receptors alpha) OR interleukin 2 receptors alpha) OR receptors alpha, interleukin-2) OR IL-2Ralpha) OR IL 2Ralpha) OR Interleukin-2Ralpha) OR interleukin 2ralpha) OR cd25 antigen) OR antigen, cd25) OR alpha-subunit, receptor, interleukin-2) OR CD25) OR IDDM10) OR IL2R) OR IMD41) OR p55) OR tcgf))) AND (((((Genetic Variation[MeSH Terms]) OR Polymorphism, Genetic[MeSH Terms]) OR Polymorphism, Single Nucleotide[MeSH Terms])) AND ((((((((((((((((((((genetic variations) OR variations, genetic) OR variation, genetic) OR diversity, genetic) OR diversities, genetic) OR genetic diversities) OR genetic diversity) OR polymorphisms, genetic) OR genetic polymorphisms) OR genetic polymorphism) OR polymorphism (genetics)) OR polymorphisms (genetics)) OR nucleotide polymorphism, single) OR nucleotide polymorphisms, single) OR polymorphisms, single nucleotide) OR single nucleotide polymorphisms) OR snps) OR single nucleotide polymorphism) OR genetic variant) OR genetic variants)) AND ((((alopecia areata[MeSH Terms]) OR alopecia circumscripta) OR alopecia totalis) OR alopecia universalis)

**Polymorphisms and risk of alopecia areata**

(((((((((((Population[MeSH Terms]) OR (((Populations) OR School Age Population) OR School Age Populations))) OR Population))) OR ((((((((Adult[MeSH Terms]) OR Aged[MeSH Terms]) OR (Aged, 80[MeSH Terms] AND over[MeSH Terms])) OR Frail Elderly[MeSH Terms]) OR Middle Aged[MeSH Terms]) OR Young Adult[MeSH Terms])) OR (((((((((((Adult, young) OR Adults, young) OR Young adults) OR Middle Age) OR Oldest Old) OR Nonagenarians) OR Nonagenarian) OR Octogenarian) OR Octogenarians) OR Centenarian) OR Centenarians))) OR ((Adolescent[MeSH Terms]) OR ((((((((Adolescents) OR Adolescence) OR Adolescent) OR Teens) OR Teenagers) OR Teenager) OR Youth) OR Youths))) OR ((((((((((Children) OR Preschool Child) OR Children, Preschool) OR Preschool Children) OR Toddler) OR Kid) OR Kids) OR Childhood) OR Pediatric) OR Infants)))) AND (((((Genetic Variation[MeSH Terms]) OR Polymorphism, Genetic[MeSH Terms]) OR Polymorphism, Single Nucleotide[MeSH Terms])) AND ((((((((((((((((((((Genetic Variations) OR Variations, Genetic) OR Variation, Genetic) OR Diversity, Genetic) OR Diversities, Genetic) OR Genetic Diversities) OR Genetic Diversity) OR Polymorphisms, Genetic) OR Genetic Polymorphisms) OR Genetic Polymorphism) OR Polymorphism (Genetics)) OR Polymorphisms (Genetics)) OR Nucleotide Polymorphism, Single) OR Nucleotide Polymorphisms, Single) OR Polymorphisms, Single Nucleotide) OR Single Nucleotide Polymorphisms) OR SNPs) OR Single Nucleotide Polymorphism) OR Genetic Variant) OR Genetic Variants))) AND ((((alopecia areata[MeSH Terms]) OR Alopecia Circumscripta) OR Alopecia totalis) OR Alopecia universalis)

***Lilacs database***

***FAS* gene and alopecia areata**

(tw:(Receptor fas OR Antígeno APO-1 OR Antígeno CD95 OR Antígeno fas OR Antígenos CD95 OR Antígenos fas OR Miembro 6 de la Superfamilia de Receptores de Factor de Necrosis Tumoral OR Receptor Fas de Muerte de Superficie Celular OR Receptor TNFRSF6 OR Receptor de muerte de la superficie celular fas OR Receptores FAS)) AND (tw:(Alopecia areata OR alopecia totalis OR alopecia universalis OR alopecia total OR alopecia universal))

***FASL* gene and alopecia areata**

(tw:(FASL OR Proteína Ligando Fas OR Antígenos CD178 OR Miembro 6 de la Superfamilia de Ligandos de Factores de Necrosis Tumoral)) AND (tw:(Alopecia areata OR alopecia totalis OR alopecia universalis OR alopecia total OR alopecia universal))

***PTPN22* gene and alopecia areata**

(tw:(Proteína Tirosina Fosfatasa no Receptora Tipo 22 OR Proteína PTPN22 OR PTPN22)) AND (tw:(Alopecia areata OR alopecia totalis OR alopecia universalis OR alopecia total OR alopecia universal))

***CTLA4* gene and alopecia areata**

(tw:(CTLA-4 Antigen OR CTLA4 protein OR CD152 OR ALPS5)) AND (tw:(Alopecia areata OR alopecia totalis OR alopecia universalis OR alopecia total OR alopecia universal))

***IL2RA* gene and alopecia areata**

(tw:(IL2RA OR Interleukin-2 Receptor alpha Subunit OR CD25 OR CD25 antigen)) AND (tw:(Alopecia areata OR alopecia totalis OR alopecia universalis OR alopecia total OR alopecia universal))

**Polymorphisms and risk of alopecia areata**

(tw:(polimorfismo OR variante genética)) AND (tw:(Alopecia areata))

**Web of Science**

***FAS* gene and alopecia areata**

TS= (Receptor, fas OR fas Antigen OR fas Antigens OR APO-1 Antigen OR APO 1 Antigen OR CD95 antigen, human OR tumor necrosis factor receptor superfamily member 6 protein, human OR FAS1 protein, human OR Fas (TNF receptor superfamily, member 6), human OR TNFRSF6 protein, human OR APO-1 OR CD95 Antigen OR TNFRSF6 Receptor OR Receptor, TNFRSF6 OR Antigens, CD95 OR fas Receptors OR Tumor Necrosis Factor Receptor Superfamily Member 6 OR Fas Cell Surface Death Receptor OR CD95 Antigens OR apo-1 antigen OR APO-1 cell surface antigen OR apoptosis antigen 1 OR apoptosis-mediating surface antigen FAS OR APT1 OR CD95 OR CD95 antigen OR Fas (TNF receptor superfamily, member 6) OR Fas AMA OR Fas antigen OR FAS1 OR FASLG receptor OR TNFRSF6 OR tumor necrosis factor receptor superfamily member) AND TS=(Alopecia Areata OR Alopecia Circumscripta OR Alopecia Totalis OR Alopecia Universalis)

***FASL* gene and alopecia areata**

TS=(Fas Ligand Protein OR FASLG protein, human OR CD95L OR Fas Ligand (FasL) OR FasL Protein
TNF Superfamily, Member 6 OR CD178 Antigen OR Antigen, CD178 OR Antigens, CD178 OR Fas Ligand OR Tumor Necrosis Factor Ligand Superfamily Member 6 OR CD178 Antigens OR CD95 Antigen Ligand OR CD95 Ligand OR APT1LG1 OR APTL OR CD95-L OR CD95L OR CD178 OR FASL OR TNFSF6) AND TS=(Alopecia Areata OR Alopecia Circumscripta OR Alopecia Totalis OR Alopecia Universalis)

***PTPN22* gene and alopecia areata**

TS= (Protein Tyrosine Phosphatase, Non Receptor Type 22 OR PTPase Lyp OR  Lymphoid Phosphatase OR Tyrosine Protein Phosphatase, Non-Receptor Type 22 OR Tyrosine Protein Phosphatase, Non Receptor Type 22 OR PTPN-22 Protein OR PTPN 22 Protein OR Protein-Tyrosine Phosphatase Lyp OR Protein Tyrosine Phosphatase Lyp OR Lyp1 PTPase OR PTPase, Lyp1 OR Lyp2; PTPase OR PTPase, Lyp2 OR protein tyrosine phosphatase, non-receptor type 8, human OR PTPN8 protein, human OR protein tyrosine phosphatase, non-receptor type 22 (lymphoid), human OR protein tyrosine phosphatase-22, human OR Lyp1 protein, human OR Lyp2 protein, human OR lymphoid phosphatase OR lymphoid-specific protein tyrosine phosphatase OR LYP OR LYP1 OR LYP2 OR PEP OR PEST-domain phosphatase OR protein tyrosine phosphatase, non-receptor type 8 OR protein tyrosine phosphatase, non-receptor type 22 (lymphoid) OR PTPN8 OR tyrosine-protein phosphatase non-receptor type 22) AND TS=(Alopecia Areata OR Alopecia Circumscripta OR Alopecia Totalis OR Alopecia Universalis)

***CTLA4* gene and alopecia areata**

TS=(CTLA-4 Antigen OR CTLA4 protein, human OR Antigen, CTLA-4 OR CTLA 4 Antigen OR Antigens, CD152 OR CD152 Antigens OR Cytotoxic T-Lymphocyte-Associated Antigen 4 OR Cytotoxic T Lymphocyte Associated Antigen 4 OR Cytotoxic T-Lymphocyte Antigen 4 OR Cytotoxic T Lymphocyte Antigen 4 OR CD152 Antigen OR Antigen, CD152 OR cytotoxic T-lymphocyte-associated protein 4, human OR CTLA-4 protein, human OR CD152 antigen, human OR ALPS5 OR CD152 OR CELIAC3 OR CTLA-4 OR GRD4 OR GSE OR IDDM12) AND TS=(Alopecia Areata OR Alopecia Circumscripta OR Alopecia Totalis OR Alopecia Universalis)

***IL2RA* gene and alopecia areata**

TS= (Interleukin 2 Receptor alpha Subunit  OR Antigens, CD25 OR CD25 Antigens OR Tac P55 Peptide OR Interleukin-2 Receptor alpha OR Interleukin 2 Receptor alpha OR Receptor alpha, Interleukin-2 OR Interleukin-2 Receptor alpha Chain OR Interleukin 2 Receptor alpha Chain OR Interleukin-2 Receptors alpha OR Interleukin 2 Receptors alpha OR Receptors alpha, Interleukin-2 OR IL-2Ralpha OR IL 2Ralpha OR Interleukin-2Ralpha OR Interleukin 2Ralpha OR CD25 Antigen OR Antigen, CD25 OR alpha-subunit, Receptor, Interleukin-2 OR interleukin 2 receptor, alpha protein, human OR CD25 antigen, human OR CD25 OR IDDM10 OR IL2R OR IMD41 OR p55 OR TCGFR) AND TS=(Alopecia Areata OR Alopecia Circumscripta OR Alopecia Totalis OR Alopecia Universalis)

**Polymorphisms and alopecia areata**

TS=(Polymorphism, Single Nucleotide OR Polymorphism, Genetic OR Genetic Variation OR Nucleotide Polymorphism, Single OR Nucleotide Polymorphisms, Single OR Polymorphisms, Single Nucleotide OR Single Nucleotide Polymorphisms OR SNPs OR Single Nucleotide Polymorphism OR Polymorphisms, Genetic OR Genetic Polymorphisms OR Genetic Polymorphism OR Polymorphism (Genetics) OR Polymorphisms (Genetics) OR Genetic Variations OR Variations, Genetic OR Variation, Genetic) AND TS=(Alopecia Areata OR Alopecia Circumscripta OR Alopecia Totalis OR Alopecia Universalis)

**Scopus database.**

***FAS* gene and alopecia areata**

( fas  OR  fas  AND antigen  OR  apo1  OR  cd95  OR  tnfrsf6  OR  fas1  OR  apt1 )  AND  ( alopecia  AND areata  OR  alopecia  AND totalis  OR  alopecia  AND universalis )

***FASL* gene and alopecia areata**

( fas  AND ligand  OR  faslg  OR  cd95l  OR  cd178  OR  apt1lg1  OR  aptl )  AND  ( alopecia  AND areata  OR  alopecia  AND circumscripta  OR  alopecia  AND totalis  OR  alopecia  AND universalis )

***PTPN22* gene and alopecia areata**

( ptpn-22 or ptpn22 or lyp or ptpn8 or lyp1 or pep ) and ( alopecia areata or alopecia circumscripta or alopecia totalis or alopecia universalis )

***CTLA4 gene and alopecia areata***

( ctla4 or ctla-4 or cd152 or celiac3 or alps5 or iddm12 or gse ) and ( alopecia areata or alopecia circumscripta or alopecia totalis or alopecia universalis )

***IL2RA gene and alopecia areata***

( IL2RA OR CD25 OR TCGFR OR IMD41 OR IL2R OR IDDM10 OR IL-2ALPHA ) AND ( Alopecia Areata OR Alopecia Circumscripta OR Alopecia Totalis OR Alopecia Universalis)

**Polymorphisms and alopecia areata**

(Genetic variant OR polymorphism OR Genetic variation OR SNP OR SNPs ) AND ( Alopecia Areata OR Alopecia Circumscripta OR Alopecia Totalis OR Alopecia Universalis)

**Embase database**

***FAS* gene and alopecia areata**

('population'/exp OR population OR populations OR 'school age population'/exp OR 'school age population' OR (('school'/exp OR school) AND ('age'/exp OR age) AND ('population'/exp OR population)) OR 'school age populations' OR (('school'/exp OR school) AND ('age'/exp OR age) AND populations) OR 'adult'/exp OR adult OR 'aged'/exp OR aged OR 'frail elderly'/exp OR 'frail elderly' OR (frail AND ('elderly'/exp OR elderly)) OR 'middle aged'/exp OR 'middle aged' OR (middle AND ('aged'/exp OR aged)) OR 'young adult'/exp OR 'young adult' OR (young AND ('adult'/exp OR adult)) OR 'adults'/exp OR adults OR 'young adults'/exp OR 'young adults' OR (young AND ('adults'/exp OR adults)) OR 'middle age'/exp OR 'middle age' OR (middle AND ('age'/exp OR age)) OR 'oldest old' OR (oldest AND old) OR 'nonagenarians'/exp OR nonagenarians OR 'nonagenarian'/exp OR nonagenarian OR 'octogenarians'/exp OR octogenarians OR 'octogenarian'/exp OR octogenarian OR 'centenarians'/exp OR centenarians OR 'centenarian'/exp OR centenarian OR 'adolescent'/exp OR adolescent OR 'adolescents'/exp OR adolescents OR 'adolescence'/exp OR adolescence OR teens OR teen OR teenagers OR 'teenager'/exp OR teenager OR 'youth'/exp OR youth OR youths OR 'child'/exp OR child OR 'child, preschool'/exp OR 'child, preschool' OR (child, AND ('preschool'/exp OR preschool)) OR 'infant'/exp OR infant OR 'children preschool' OR (('children'/exp OR children) AND ('preschool'/exp OR preschool)) OR 'child preschool'/exp OR 'child preschool' OR (('child'/exp OR child) AND ('preschool'/exp OR preschool)) OR 'children'/exp OR children OR 'toddler'/exp OR toddler OR kid OR kids OR 'childhood'/exp OR childhood OR 'pediatric'/exp OR pediatric OR 'infants'/exp OR infants) AND ('fas receptor'/exp OR 'fas receptor' OR (fas AND ('receptor'/exp OR receptor)) OR 'fas protein, human' OR (fas AND protein, AND ('human'/exp OR human))) AND ('alopecia areata'/exp OR 'alopecia areata' OR (('alopecia'/exp OR alopecia) AND areata) OR 'alopecia circumscripta'/exp OR 'alopecia circumscripta' OR (('alopecia'/exp OR alopecia) AND circumscripta) OR 'alopecia totalis'/exp OR 'alopecia totalis' OR (('alopecia'/exp OR alopecia) AND totalis) OR 'alopecia universalis'/exp OR 'alopecia universalis' OR (('alopecia'/exp OR alopecia) AND universalis))

***FASL* gene and alopecia areata**

('population'/exp OR population OR populations OR 'school age population'/exp OR 'school age population' OR (('school'/exp OR school) AND ('age'/exp OR age) AND ('population'/exp OR population)) OR 'school age populations' OR (('school'/exp OR school) AND ('age'/exp OR age) AND populations) OR 'adult'/exp OR adult OR 'aged'/exp OR aged OR 'frail elderly'/exp OR 'frail elderly' OR (frail AND ('elderly'/exp OR elderly)) OR 'middle aged'/exp OR 'middle aged' OR (middle AND ('aged'/exp OR aged)) OR 'young adult'/exp OR 'young adult' OR (young AND ('adult'/exp OR adult)) OR 'adults'/exp OR adults OR 'young adults'/exp OR 'young adults' OR (young AND ('adults'/exp OR adults)) OR 'middle age'/exp OR 'middle age' OR (middle AND ('age'/exp OR age)) OR 'oldest old' OR (oldest AND old) OR 'nonagenarians'/exp OR nonagenarians OR 'nonagenarian'/exp OR nonagenarian OR 'octogenarians'/exp OR octogenarians OR 'octogenarian'/exp OR octogenarian OR 'centenarians'/exp OR centenarians OR 'centenarian'/exp OR centenarian OR 'adolescent'/exp OR adolescent OR 'adolescents'/exp OR adolescents OR 'adolescence'/exp OR adolescence OR teens OR teen OR teenagers OR 'teenager'/exp OR teenager OR 'youth'/exp OR youth OR youths OR 'child'/exp OR child OR 'child, preschool'/exp OR 'child, preschool' OR (child, AND ('preschool'/exp OR preschool)) OR 'infant'/exp OR infant OR 'children preschool' OR (('children'/exp OR children) AND ('preschool'/exp OR preschool)) OR 'child preschool'/exp OR 'child preschool' OR (('child'/exp OR child) AND ('preschool'/exp OR preschool)) OR 'children'/exp OR children OR 'toddler'/exp OR toddler OR kid OR kids OR 'childhood'/exp OR childhood OR 'pediatric'/exp OR pediatric OR 'infants'/exp OR infants) AND ('fas ligand protein'/exp OR 'fas ligand protein' OR (fas AND ('ligand'/exp OR ligand) AND ('protein'/exp OR protein)) OR 'faslg protein,human' OR (faslg AND protein,human)) AND ('alopecia areata'/exp OR 'alopecia areata' OR (('alopecia'/exp OR alopecia) AND areata) OR 'alopecia circumscripta'/exp OR 'alopecia circumscripta' OR (('alopecia'/exp OR alopecia) AND circumscripta) OR 'alopecia totalis'/exp OR 'alopecia totalis' OR (('alopecia'/exp OR alopecia) AND totalis) OR 'alopecia universalis'/exp OR 'alopecia universalis' OR (('alopecia'/exp OR alopecia) AND universalis))

***IL2RA* and alopecia areata**

('population'/exp OR population OR populations OR 'school age population'/exp OR 'school age population' OR (('school'/exp OR school) AND ('age'/exp OR age) AND ('population'/exp OR population)) OR 'school age populations' OR (('school'/exp OR school) AND ('age'/exp OR age) AND populations) OR 'adult'/exp OR adult OR 'aged'/exp OR aged OR 'frail elderly'/exp OR 'frail elderly' OR (frail AND ('elderly'/exp OR elderly)) OR 'middle aged'/exp OR 'middle aged' OR (middle AND ('aged'/exp OR aged)) OR 'young adult'/exp OR 'young adult' OR (young AND ('adult'/exp OR adult)) OR 'adults'/exp OR adults OR 'young adults'/exp OR 'young adults' OR (young AND ('adults'/exp OR adults)) OR 'middle age'/exp OR 'middle age' OR (middle AND ('age'/exp OR age)) OR 'oldest old' OR (oldest AND old) OR 'nonagenarians'/exp OR nonagenarians OR 'nonagenarian'/exp OR nonagenarian OR 'octogenarians'/exp OR octogenarians OR 'octogenarian'/exp OR octogenarian OR 'centenarians'/exp OR centenarians OR 'centenarian'/exp OR centenarian OR 'adolescent'/exp OR adolescent OR 'adolescents'/exp OR adolescents OR 'adolescence'/exp OR adolescence OR teens OR teen OR teenagers OR 'teenager'/exp OR teenager OR 'youth'/exp OR youth OR youths OR 'child'/exp OR child OR 'child, preschool'/exp OR 'child, preschool' OR (child, AND ('preschool'/exp OR preschool)) OR 'infant'/exp OR infant OR 'children preschool' OR (('children'/exp OR children) AND ('preschool'/exp OR preschool)) OR 'child preschool'/exp OR 'child preschool' OR (('child'/exp OR child) AND ('preschool'/exp OR preschool)) OR 'children'/exp OR children OR 'toddler'/exp OR toddler OR kid OR kids OR 'childhood'/exp OR childhood OR 'pediatric'/exp OR pediatric OR 'infants'/exp OR infants) AND ('interleukin-2 receptor alpha subunit'/exp OR 'interleukin-2 receptor alpha subunit' OR (('interleukin 2'/exp OR 'interleukin 2') AND ('receptor'/exp OR receptor) AND ('alpha'/exp OR alpha) AND subunit) OR 'il2ra protein,human' OR (il2ra AND protein,human)) AND ('alopecia areata'/exp OR 'alopecia areata' OR (('alopecia'/exp OR alopecia) AND areata) OR 'alopecia circumscripta'/exp OR 'alopecia circumscripta' OR (('alopecia'/exp OR alopecia) AND circumscripta) OR 'alopecia totalis'/exp OR 'alopecia totalis' OR (('alopecia'/exp OR alopecia) AND totalis) OR 'alopecia universalis'/exp OR 'alopecia universalis' OR (('alopecia'/exp OR alopecia) AND universalis))

***CTLA4* and alopecia areata**

('population'/exp OR population OR populations OR 'school age population'/exp OR 'school age population' OR (('school'/exp OR school) AND ('age'/exp OR age) AND ('population'/exp OR population)) OR 'school age populations' OR (('school'/exp OR school) AND ('age'/exp OR age) AND populations) OR 'adult'/exp OR adult OR 'aged'/exp OR aged OR 'frail elderly'/exp OR 'frail elderly' OR (frail AND ('elderly'/exp OR elderly)) OR 'middle aged'/exp OR 'middle aged' OR (middle AND ('aged'/exp OR aged)) OR 'young adult'/exp OR 'young adult' OR (young AND ('adult'/exp OR adult)) OR 'adults'/exp OR adults OR 'young adults'/exp OR 'young adults' OR (young AND ('adults'/exp OR adults)) OR 'middle age'/exp OR 'middle age' OR (middle AND ('age'/exp OR age)) OR 'oldest old' OR (oldest AND old) OR 'nonagenarians'/exp OR nonagenarians OR 'nonagenarian'/exp OR nonagenarian OR 'octogenarians'/exp OR octogenarians OR 'octogenarian'/exp OR octogenarian OR 'centenarians'/exp OR centenarians OR 'centenarian'/exp OR centenarian OR 'adolescent'/exp OR adolescent OR 'adolescents'/exp OR adolescents OR 'adolescence'/exp OR adolescence OR teens OR teen OR teenagers OR 'teenager'/exp OR teenager OR 'youth'/exp OR youth OR youths OR 'child'/exp OR child OR 'child, preschool'/exp OR 'child, preschool' OR (child, AND ('preschool'/exp OR preschool)) OR 'infant'/exp OR infant OR 'children preschool' OR (('children'/exp OR children) AND ('preschool'/exp OR preschool)) OR 'child preschool'/exp OR 'child preschool' OR (('child'/exp OR child) AND ('preschool'/exp OR preschool)) OR 'children'/exp OR children OR 'toddler'/exp OR toddler OR kid OR kids OR 'childhood'/exp OR childhood OR 'pediatric'/exp OR pediatric OR 'infants'/exp OR infants) AND ('ctla-4 antigen'/exp OR 'ctla-4 antigen' OR (('ctla 4'/exp OR 'ctla 4') AND ('antigen'/exp OR antigen)) OR 'ctla4 protein,human' OR (('ctla4'/exp OR ctla4) AND protein,human)) AND ('alopecia areata'/exp OR 'alopecia areata' OR (('alopecia'/exp OR alopecia) AND areata) OR 'alopecia circumscripta'/exp OR 'alopecia circumscripta' OR (('alopecia'/exp OR alopecia) AND circumscripta) OR 'alopecia totalis'/exp OR 'alopecia totalis' OR (('alopecia'/exp OR alopecia) AND totalis) OR 'alopecia universalis'/exp OR 'alopecia universalis' OR (('alopecia'/exp OR alopecia) AND universalis))

***PTPN22* gene and alopecia areata**

('population'/exp OR population OR populations OR 'school age population'/exp OR 'school age population' OR (('school'/exp OR school) AND ('age'/exp OR age) AND ('population'/exp OR population)) OR 'school age populations' OR (('school'/exp OR school) AND ('age'/exp OR age) AND populations) OR 'adult'/exp OR adult OR 'aged'/exp OR aged OR 'frail elderly'/exp OR 'frail elderly' OR (frail AND ('elderly'/exp OR elderly)) OR 'middle aged'/exp OR 'middle aged' OR (middle AND ('aged'/exp OR aged)) OR 'young adult'/exp OR 'young adult' OR (young AND ('adult'/exp OR adult)) OR 'adults'/exp OR adults OR 'young adults'/exp OR 'young adults' OR (young AND ('adults'/exp OR adults)) OR 'middle age'/exp OR 'middle age' OR (middle AND ('age'/exp OR age)) OR 'oldest old' OR (oldest AND old) OR 'nonagenarians'/exp OR nonagenarians OR 'nonagenarian'/exp OR nonagenarian OR 'octogenarians'/exp OR octogenarians OR 'octogenarian'/exp OR octogenarian OR 'centenarians'/exp OR centenarians OR 'centenarian'/exp OR centenarian OR 'adolescent'/exp OR adolescent OR 'adolescents'/exp OR adolescents OR 'adolescence'/exp OR adolescence OR teens OR teen OR teenagers OR 'teenager'/exp OR teenager OR 'youth'/exp OR youth OR youths OR 'child'/exp OR child OR 'child, preschool'/exp OR 'child, preschool' OR (child, AND ('preschool'/exp OR preschool)) OR 'infant'/exp OR infant OR 'children preschool' OR (('children'/exp OR children) AND ('preschool'/exp OR preschool)) OR 'child preschool'/exp OR 'child preschool' OR (('child'/exp OR child) AND ('preschool'/exp OR preschool)) OR 'children'/exp OR children OR 'toddler'/exp OR toddler OR kid OR kids OR 'childhood'/exp OR childhood OR 'pediatric'/exp OR pediatric OR 'infants'/exp OR infants) AND ('protein tyrosine phosphatase, non-receptor type 22'/exp OR 'protein tyrosine phosphatase, non-receptor type 22' OR (('protein'/exp OR protein) AND ('tyrosine'/exp OR tyrosine) AND phosphatase, AND 'non receptor' AND type AND 22) OR 'ptpn22 protein,human' OR (ptpn22 AND protein,human)) AND ('alopecia areata'/exp OR 'alopecia areata' OR (('alopecia'/exp OR alopecia) AND areata) OR 'alopecia circumscripta'/exp OR 'alopecia circumscripta' OR (('alopecia'/exp OR alopecia) AND circumscripta) OR 'alopecia totalis'/exp OR 'alopecia totalis' OR (('alopecia'/exp OR alopecia) AND totalis) OR 'alopecia universalis'/exp OR 'alopecia universalis' OR (('alopecia'/exp OR alopecia) AND universalis))

**Polymorphisms and alopecia areata**

('population'/exp OR population OR populations OR 'school age population'/exp OR 'school age population' OR (('school'/exp OR school) AND ('age'/exp OR age) AND ('population'/exp OR population)) OR 'school age populations' OR (('school'/exp OR school) AND ('age'/exp OR age) AND populations) OR 'adult'/exp OR adult OR 'aged'/exp OR aged OR 'frail elderly'/exp OR 'frail elderly' OR (frail AND ('elderly'/exp OR elderly)) OR 'middle aged'/exp OR 'middle aged' OR (middle AND ('aged'/exp OR aged)) OR 'young adult'/exp OR 'young adult' OR (young AND ('adult'/exp OR adult)) OR 'adults'/exp OR adults OR 'young adults'/exp OR 'young adults' OR (young AND ('adults'/exp OR adults)) OR 'middle age'/exp OR 'middle age' OR (middle AND ('age'/exp OR age)) OR 'oldest old' OR (oldest AND old) OR 'nonagenarians'/exp OR nonagenarians OR 'nonagenarian'/exp OR nonagenarian OR 'octogenarians'/exp OR octogenarians OR 'octogenarian'/exp OR octogenarian OR 'centenarians'/exp OR centenarians OR 'centenarian'/exp OR centenarian OR 'adolescent'/exp OR adolescent OR 'adolescents'/exp OR adolescents OR 'adolescence'/exp OR adolescence OR teens OR teen OR teenagers OR 'teenager'/exp OR teenager OR 'youth'/exp OR youth OR youths OR 'child'/exp OR child OR 'child, preschool'/exp OR 'child, preschool' OR (child, AND ('preschool'/exp OR preschool)) OR 'infant'/exp OR infant OR 'children preschool' OR (('children'/exp OR children) AND ('preschool'/exp OR preschool)) OR 'child preschool'/exp OR 'child preschool' OR (('child'/exp OR child) AND ('preschool'/exp OR preschool)) OR 'children'/exp OR children OR 'toddler'/exp OR toddler OR kid OR kids OR 'childhood'/exp OR childhood OR 'pediatric'/exp OR pediatric OR 'infants'/exp OR infants) AND ('polymorphism,single nucleotide'/exp OR 'polymorphism,single nucleotide' OR (polymorphism,single AND ('nucleotide'/exp OR nucleotide)) OR 'polymorphism,genetic'/exp OR polymorphism,genetic OR 'genetic variation'/exp OR 'genetic variation' OR (('genetic'/exp OR genetic) AND ('variation'/exp OR variation)) OR 'nucleotide polymorphism, single' OR (('nucleotide'/exp OR nucleotide) AND polymorphism, AND single) OR 'nucleotide polymorphisms, single' OR (('nucleotide'/exp OR nucleotide) AND polymorphisms, AND single) OR 'polymorphisms, single nucleotide' OR (polymorphisms, AND single AND ('nucleotide'/exp OR nucleotide)) OR 'single nucleotide polymorphisms'/exp OR 'single nucleotide polymorphisms' OR (single AND ('nucleotide'/exp OR nucleotide) AND polymorphisms) OR 'snps'/exp OR snps OR 'single nucleotide polymorphism'/exp OR 'single nucleotide polymorphism' OR (single AND ('nucleotide'/exp OR nucleotide) AND ('polymorphism'/exp OR polymorphism)) OR 'polymorphisms, genetic' OR (polymorphisms, AND ('genetic'/exp OR genetic)) OR 'genetic polymorphisms' OR (('genetic'/exp OR genetic) AND polymorphisms) OR 'genetic polymorphism'/exp OR 'genetic polymorphism' OR (('genetic'/exp OR genetic) AND ('polymorphism'/exp OR polymorphism)) OR (('polymorphism'/exp OR polymorphism) AND ('genetics'/exp OR genetics)) OR (polymorphisms AND ('genetics'/exp OR genetics)) OR 'genetic variations' OR (('genetic'/exp OR genetic) AND variations) OR 'variations, genetic' OR (variations, AND ('genetic'/exp OR genetic)) OR 'variation, genetic'/exp OR 'variation, genetic' OR (variation, AND ('genetic'/exp OR genetic))) AND ('alopecia areata'/exp OR 'alopecia areata' OR (('alopecia'/exp OR alopecia) AND areata) OR 'alopecia circumscripta'/exp OR 'alopecia circumscripta' OR (('alopecia'/exp OR alopecia) AND circumscripta) OR 'alopecia totalis'/exp OR 'alopecia totalis' OR (('alopecia'/exp OR alopecia) AND totalis) OR 'alopecia universalis'/exp OR 'alopecia universalis' OR (('alopecia'/exp OR alopecia) AND universalis))

**Google scholar**

***FAS* gene and alopecia areata**

FAS RECEPTOR OR FAS OR CD95 OR APO-1 "ALOPECIA AREATA"

***FASL* gene and alopecia areata**

FAS LIGAND OR FASL OR CD178 "ALOPECIA AREATA"

***PTPN22* gene and alopecia areata**

PTPN22 OR PROTEIN TYROSINE PHOSPHATASE NON RECEPTOR TYPE 22 "ALOPECIA AREATA"

***CTLA4* gene and alopecia areata**

CTLA4 OR CTLA-4 OR CD152 OR CTLA4 ANTIGEN  "ALOPECIA AREATA"

***IL2RA* gene and alopecia areata**

IL2RA OR INTERLEUKIN-2 RECEPTOR ALPHA SUBUNIT OR CD25 "ALOPECIA AREATA"

**Polymorphisms and alopecia areata**

GENETIC VARIANT OR POLYMORPHISM OR SNP "ALOPECIA AREATA"

**OPENGREY**

***FAS* gene and alopecia areata**

(FAS RECEPTOR OR FAS OR CD95 OR APO-1) AND (ALOPECIA AREATA)

***FASL* gene and alopecia areata**

(FAS LIGAND OR FASL OR CD178) AND (ALOPECIA AREATA)

***PTPN22* gene and alopecia areata**

(PTPN22 OR PROTEIN TYROSINE PHOSPHATASE NON-RECEPTOR TYPE 22) AND (ALOPECIA AREATA)

***CTLA4* gene and alopecia areata**

(CTLA4 OR CTLA-4 OR CD152 OR CTLA4 ANTIGEN) AND ALOPECIA AREATA

***IL2RA* gene and alopecia areata**

(IL2RA OR INTERLEUKIN-2 RECEPTOR ALPHA SUBUNIT OR CD25) AND ALOPECIA AREATA

**Polymorphisms and alopecia areata**

(GENETIC VARIANT OR POLYMORPHISM OR SNP) AND (ALOPECIA AREATA)
