# Supplementary material for: Effect of PTPN22, FAS/FASL, IL2RA and CTLA4 genetic polymorphisms on the risk of developing alopecia areata: A systematic review of the literature and meta-analysis
Source: PLoS One. 2021 Nov 4;16(11):e0258499. doi: 10.1371/journal.pone.0258499 (PMC8568157; doi:10.1371/journal.pone.0258499)
Supplement: S3 File — (DOCX) [file pone.0258499.s003.docx]

**Quality of evidence assessment using GRADEpro tool**

**Table 1.** ***PTPN22* rs2476601 allele *T* vs PTPN22 rs2476601 allele *C***

| **Certainty assessment** | | | | | | | **#patients** | | **Effect** | | **Certainty** | **Importance** |
| --- | --- | --- | --- | --- | --- | --- | --- | --- | --- | --- | --- | --- |
| **#of studies** | **Study design** | **Risk of bias** | **Inconsistency** | **Indirect evidence** | **Impressicion** | **Other considerations** | **PTPN22 RS2476601 ALLELE T** | **PTPN22 RS2476601 ALLELE C** | **Relative (95% CI)** | **Absolute (95% CI)** |  |  |
| ALOPECIA AREATA (evaluated by: genetic sequencing) | | | | | | | | | | | | |
| 7 | Observational studies | no es serio | serious ^a^ | Not serious | not serious | none | 2584 cases 3664 Controls | | **OR 1.49** (1.13 a 1.95) | - | MODERATE | CRÍTICAL |
|  |  |  |  |  |  |  | - | 0.0% |  | **0 fewer per 1000** (de 0 fewer per 0 fewer) |  |  |

**CI:** confidence interval; **OR:** odds ratio

**Table 2 *PTPN22* rs2476601 genotype *CT* vs *PTPN22* rs2476601 genotype *CC***

| **Certainty assessment** | | | | | | | **Certainty assessment** | | **Certainty assessment** | | **Certainty assessment** | **Importance** |
| --- | --- | --- | --- | --- | --- | --- | --- | --- | --- | --- | --- | --- |
| **# of studies** | **Study design** | **Risk of bias** | **Inconsistency** | **Indirectness** | **Imprecision** | **Other considerations** | **PTPN22 RS2476601 GENOTYPE CT** | **PTPN22 RS247660 GENOTYPE CC** | **Relative (95% CI)** | **Absolute (95% CI)** |  |  |
| ALOPECIA AREATA (evaluated by: genetic sequencing) | | | | | | | | | | | | |
| 6 | Observational studies | Not serious | Not serious | not serious | not serious | none | 1232 cases 1832 Controls | | **OR 1.44** (1.18 a 1.76) | - | HIGH | CRÍTICAL |
|  |  |  |  |  |  |  | - | 0.0% |  | **0 fewer por 1000** (0 fewer to 0 fewer) |  |  |

**CI:** confidence interval; **OR:** odds ratio

**Table 3*- PTPN22 rs*2476601 genotype *CT*+*TT* vs *PTPN22* rs2476601 genotype *CC***

| **Certainty assessment** | | | | | | | **Certainty assessment** | | **Effect** | | **Certainty** | **Importance** |
| --- | --- | --- | --- | --- | --- | --- | --- | --- | --- | --- | --- | --- |
| **# of studies** | **Study design** | **Risk of bias** | **Inconsistency** | **Indirectness** | **Imprecision** | **Other considerations** | **PTPN22 RS2476601 GENOTIPE CT+TT** | **PTPN22 RS2476601 GENOTIPE CC** | **Relativo (95% CI)** | **Absolute (95% CI)** |  |  |
| ALOPECIA AREATA (evaluated by : genetic sequencing) | | | | | | | | | | | | |
| 6 | Observational studies | Not serious | Not serious | Not serious | Not serious | none | 1232 cases 1832 Controls | | **OR 1.43** (1.18 a 1.76) | - | HIGH | CRÍTICAL |
|  |  |  |  |  |  |  | - | 0.0% |  | **0 fewer per 1000** (0 fewer to 0 fewer) |  |  |

**CI:** confidence interval; **OR:** odds ratio
