# Supplementary material for: Effect of PTPN22, FAS/FASL, IL2RA and CTLA4 genetic polymorphisms on the risk of developing alopecia areata: A systematic review of the literature and meta-analysis
Source: PLoS One. 2021 Nov 4;16(11):e0258499. doi: 10.1371/journal.pone.0258499 (PMC8568157; doi:10.1371/journal.pone.0258499)

**Forest plot performed for the *PTPN22* gene*.***

*PTPN22* - Allelic model (T vs C) per subgroup of control selection.


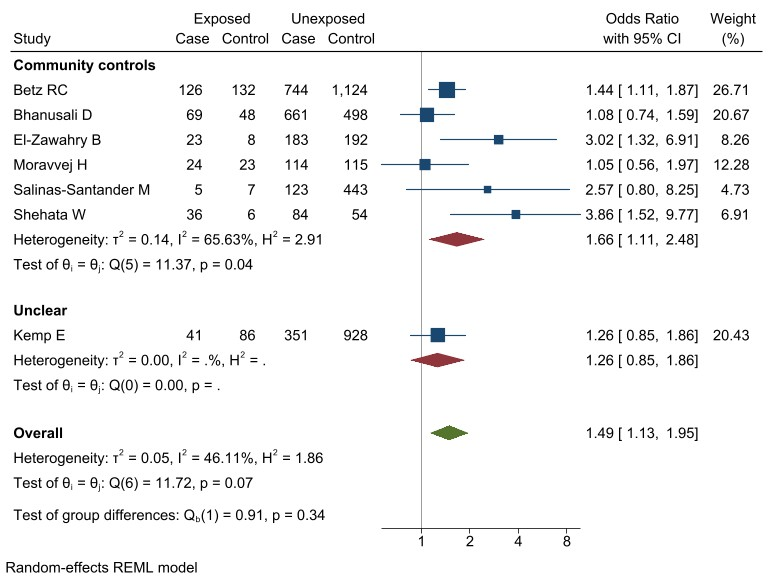

Supplement: S4 File — PTPN22. (DOCX) [file pone.0258499.s004.docx]
