# Supplementary figures and images for: Effect of PTPN22, FAS/FASL, IL2RA and CTLA4 genetic polymorphisms on the risk of developing alopecia areata: A systematic review of the literature and meta-analysis
Source: PLoS One. 2021 Nov 4;16(11):e0258499. doi: 10.1371/journal.pone.0258499 (PMC8568157; doi:10.1371/journal.pone.0258499)

**Forest plot performed for the *FAS* gene.**

*FAS* - Allelic model (*G* vs *A*)

**
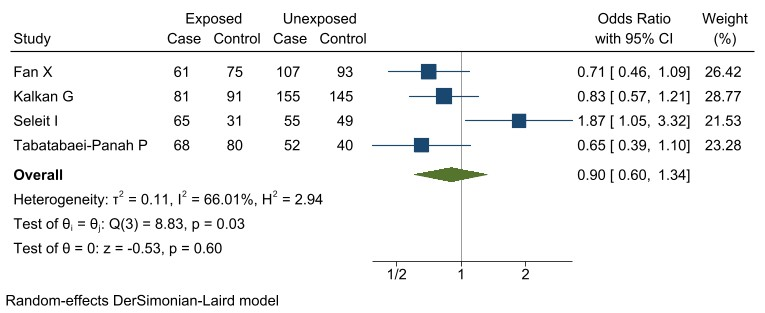
**

Supplement: S5 File — (DOCX) [file pone.0258499.s005.docx]

**Forest plot performed for the *FASL* gene.**

*FASL-* Allelic model (*G* vs *A*)**
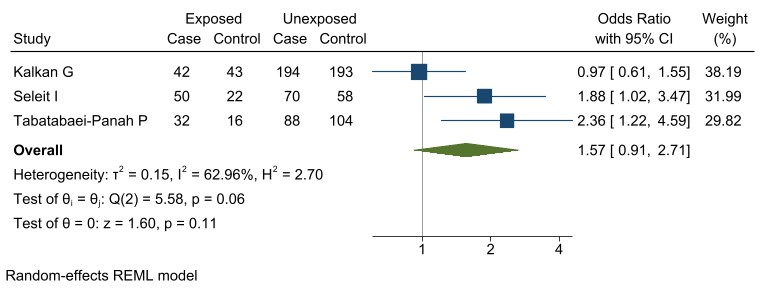
**

Supplement: S6 File — (DOCX) [file pone.0258499.s006.docx]

**Forest plot performed for *CTLA4* gene.**

*CTLA4.* Allelic model (*G* vs *A*)**
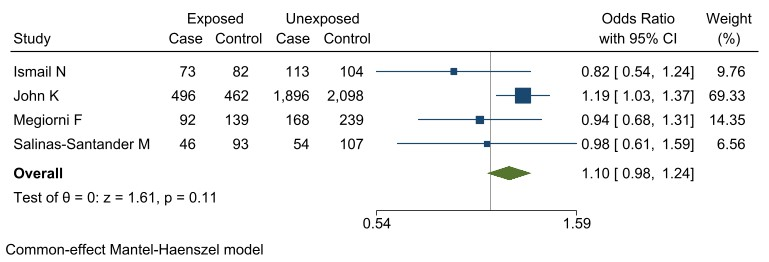
**

Supplement: S7 File — (DOCX) [file pone.0258499.s007.docx]
