# Supplementary material for: Effect of PTPN22, FAS/FASL, IL2RA and CTLA4 genetic polymorphisms on the risk of developing alopecia areata: A systematic review of the literature and meta-analysis
Source: PLoS One. 2021 Nov 4;16(11):e0258499. doi: 10.1371/journal.pone.0258499 (PMC8568157; doi:10.1371/journal.pone.0258499)
Supplement: Attachment — Submitted filename: for plos one.docx [file pone.0258499.s008.docx]

- The manuscript generally good.

- There are many language and grammar errors and need to be edited correctly.

- **There are minor revisions as follow:**

- Line 65, the word its should be their.

- Line 66, function of what?

- Line 77, the word report should be reported

- Line 84, (Or=0 .28, 95% CI 0.09-0.82) is the same for allele A (rs 231775) and G (rs 3087243)??

- Line 92, its should be their.

- Line 109, Gen is the abbreviation of what?

- Line 170, rs of FAS gene is written incorrectly.

- Line 171, the authors should write rs of CTLA4 gene.

- In statistical analysis part and other parts, the word analyzes is wrong. This word is verb and not noun.

- In table 3, why the authors write allele ½ in the head of study information column and write allele 2 in head of cases column.

- In line 221, Rs should be rs

- Replace each word alele by word allele.

- In line 280, LYP ptn, the abbreviation of what ?

- Demonstrate the abbreviated LCK protein and CSK terms.

- The paragraph included in lines 286 to 290 in discussion need more clarification.

- Line 366, C>T or T>C. it should T>C.

- Line 384 in discussion part, p values results not significant as authors determined.

- Line 434, the word a priori word is wrong.
