# Supplementary material for: Effect of PTPN22, FAS/FASL, IL2RA and CTLA4 genetic polymorphisms on the risk of developing alopecia areata: A systematic review of the literature and meta-analysis
Source: PLoS One. 2021 Nov 4;16(11):e0258499. doi: 10.1371/journal.pone.0258499 (PMC8568157; doi:10.1371/journal.pone.0258499)
Supplement: Attachment — Submitted filename: Response to Reviewers.docx [file pone.0258499.s009.docx]

Dear editor

Thank for the review.

The manuscript generally good.

- There are many language and grammar errors and need to be edited correctly.

**Answer: the manuscript has been fully corrected**

- **There are minor revisions as follow:**

- Line 65, the word its should be their.

**Answer: the correction has been made**

- Line 66, function of what?

**Answer: the sentence has been changed and explained**

- Line 77, the word report should be reported

**Answer: the word has been corrected**

- Line 84, (Or=0 .28, 95% CI 0.09-0.82) is the same for allele A (rs 231775) and G (rs 3087243)?.

Answer**: In the paragraph, the OR for the haplotype containing these two alleleshas been estimated. The sentence was corrected**

- Line 92, its should be their.

**Answer: error has been corrected**

- Line 109, Gen is the abbreviation of what?:

**Answer: the correct word is Gene; the word has been corrected**

- Line 170, rs of FAS gene is written incorrectly.

**Answer: rs for the FAS gene has been corrected**

- Line 171, the authors should write rs of CTLA4 gene.

**Answer: CTLA4 gene rs has been corrected**

- In statistical analysis part and other parts, the word analyzes is wrong. This word is verb and not noun.

Answer: **the word analyzes has been corrected, it has been changed by analysis.**

- In table 3, why the authors write allele ½ in the head of study information column and write allele 2 in head of cases column.

**Answer: allele number 1 alluded to the major allele and allele number 2 to the minor allele. The correct name has been corrected in the table.**

- In line 221, Rs should be rs

**Answer: the correction has been made**

- Replace each word alele by word allele.

**Answer: the correction has been made**

- In line 280, LYP ptn, the abbreviation of what?

**Answer: the abbreviation has been described in the paragraph**

- Demonstrate the abbreviated LCK protein and CSK terms.

**Answer: the abbreviations has been described in the paragraph**

- The paragraph included in lines 286 to 290 in discussion need more clarification.

**Answer: The information in the paragraph has been expanded.**

- Line 366, C>T or T>C. it should T>C.

Answer: **the correct manner is T>C. It has been corrected.**

- Line 384 in discussion part, p values results not significant as authors determined.

Answer: **the information in the paragraph has been corrected**

- Line 434, the word a priori word is wrong.

Answer: **the paragraph was restructured**

**Reviewers' comments:**

Reviewer's Responses to Questions

**Comments to the Author**

1. Is the manuscript technically sound, and do the data support the conclusions?

The manuscript must describe a technically sound piece of scientific research with data that supports the conclusions. Experiments must have been conducted rigorously, with appropriate controls, replication, and sample sizes. The conclusions must be drawn appropriately based on the data presented.

Reviewer #1: Yes

Reviewer #2: Yes

2. Has the statistical analysis been performed appropriately and rigorously?

Reviewer #1: Yes

Reviewer #2: Yes

3. Have the authors made all data underlying the findings in their manuscript fully available?

The [PLOS Data policy](http://www.plosone.org/static/policies.action#sharing) requires authors to make all data underlying the findings described in their manuscript fully available without restriction, with rare exception (please refer to the Data Availability Statement in the manuscript PDF file). The data should be provided as part of the manuscript or its supporting information, or deposited to a public repository. For example, in addition to summary statistics, the data points behind means, medians and variance measures should be available. If there are restrictions on publicly sharing data—e.g. participant privacy or use of data from a third party—those must be specified.

Reviewer #1: Yes

Reviewer #2: Yes

4. Is the manuscript presented in an intelligible fashion and written in standard English?

PLOS ONE does not copyedit accepted manuscripts, so the language in submitted articles must be clear, correct, and unambiguous. Any typographical or grammatical errors should be corrected at revision, so please note any specific errors here.

Reviewer #1: No

Reviewer #2: No

**Answer: The manuscript was revised again in order to correct English grammar errors.**

5. Review Comments to the Author

Reviewer #1: Authors have investigated the polymorphisms of the candidate genes in susceptibility to alopecia areata in a meta-analysis study. They have indicated that rs2476601 variation in the PTPN22 gene is a risk factor for the development of alopecia areata. The authors did a nice job to summarize the previous studies regarding the influence of genetic alteration in susceptibility to alopecia areata. I have only some minor comments:

- Although the discussion part is categorized to explain all the variations in several populations with different ethnicity, this part needs to be improved by making a short conclusion at the end of each section.

**Answer**: **the explanation was supplemented at the end of each paragraph**

Only explaining the results of the previous studies is not enough for a systematic review of the literature. In the present form, the authors mostly stated the results which some of which are significant in the specific populations. However, at the end of the study, they conclude that only a variation in the PTPN22 gene is associated with the disease.

**Answer: According to the analysis carried out, it was found that only the PTPN22 gene has statistical significance. Regarding to the other genes, some studies show statistical significance, however when all the studies are analyzed together (as a meta analysis) and the quality of the studies is evaluated, their role in the risk of developing alopecia cannot be demonstrated.**

- The authors stated in the title of the study "Effect of polymorphisms on PTPN22, FAS/FASL, IL2RA and CTLA4 genes on the risk of developing alopecia areata". Are the authors are evaluating the influence of the polymorphisms on the genes (it seems that there is no data available reporting gene alteration) or the disease development. If the second is correct (which it seems that this is the case), the title needs to be revised.

**Answer: The title was corrected. We evaluated the influence of the polymorphisms on the alopecia areata development.**

- The English language needs to seriously be improved.

**Answer: The manuscript was revised again in order to correct English grammar errors.**

_________________________________________________

Reviewer #2: the manuscript generally good, I did not find dual publication. I detected that the research ethics are provided in the manuscript. the manuscript need English and good grammar editing, it should revised by native English speaker.

**Answer: The manuscript was revised again in order to correct English grammar errors.**
